# Systematic review and meta-analysis of intravenous iron therapy for patients with heart failure and iron deficiency

**DOI:** 10.1038/s41591-025-03671-1

**Published:** 2025-03-30

**Authors:** Stefan D. Anker, Mahir Karakas, Robert J. Mentz, Piotr Ponikowski, Javed Butler, Muhammad Shahzeb Khan, Khawaja M. Talha, Paul R. Kalra, Adrian F. Hernandez, Hillary Mulder, Frank W. Rockhold, Marius Placzek, Christian Röver, John G. F. Cleland, Tim Friede

**Affiliations:** 1 Deutsches Herzzentrum der Charité, Campus Virchow Klinikum, Berlin, Germany; 2 Institute of Health Centre for Regenerative Therapies (BCRT), German Centre for Cardiovascular Research (DZHK), Charité Universitätsmedizin, Berlin, Germany; 3 Department of Intensive Care Medicine, University Medical Centre Hamburg-Eppendorf, Hamburg, Germany; 4 German Centre for Cardiovascular Research (DZHK), Hamburg, Germany; 5 Duke University Medical Center and Duke Clinical Research Institute, Durham, NC USA; 6 Institute of Heart Diseases, Medical University and University Hospital, Wroclaw, Poland; 7 Department of Medicine, University of Mississippi Medical Center, Jackson, MS USA; 8 Baylor Scott and White Research Institute, Dallas, TX USA; 9 Baylor Scott and White Health: The Heart Hospitals, Plano, TX USA; 10 Department of Cardiology, Loyola University Medical Center, Maywood, IL USA; 11 Department of Cardiology, Portsmouth Hospitals University NHS Trust, Portsmouth, UK; 12 Department of Medical Statistics, University Medical Centre Göttingen, Göttingen, Germany; 13 Department of Cardiology, British Heart Foundation Cardiovascular Research Centre, School of Cardiovascular and Metabolic Health, University of Glasgow, Glasgow, UK; 14 German Centre for Cardiovascular Research (DZHK), Göttingen, Germany

**Keywords:** Drug therapy, Outcomes research

## Abstract

Uncertainty remains about the effect of intravenous (i.v.) iron on outcomes for heart failure (HF) with iron deficiency. In the present study, we summarize the efficacy and safety of i.v. iron from six trials (FAIR-HF, CONFIRM-HF, AFFIRM-AHF, IRONMAN, HEART-FID and FAIR-HF2), including 7,175 patients. In comparison to prior analyses, this meta-analysis added new data from FAIR-HF2, used a harmonized and robust Bayesian approach and included individual participant data from five trials. Patients assigned to i.v. iron, compared with those assigned to placebo, had lower rates for the composite endpoint of recurrent HF hospitalizations and cardiovascular mortality at 12 months (risk ratio (RR) = 0.72 (95% confidence interval (CI) = 0.55–0.89)) and for the complete length of follow-up (RR = 0.81 (95% CI = 0.63–0.97)). Each component of the primary endpoint contributed to the beneficial effect of i.v. iron at both 12 months and the complete length of follow-up: recurrent HF hospitalizations (RR = 0.69 (95% CI = 0.48–0.88) and RR = 0.78 (95% CI = 0.55–0.98), respectively) and cardiovascular mortality (hazard ratio (HR) = 0.80 (95% CI = 0.61–1.03) and HR = 0.87 (95% CI = 0.73–1.04), respectively). All-cause mortality at 12 months and for the complete length of follow-up (HR = 0.82 (95% CI = 0.65–1.03)) and HR = 0.92 (95% CI = 0.80–1.07), respectively, indicated the overall safety of i.v. iron treatment. Treatment effects were greatest in the first year after randomization when the doses of i.v. iron provided are highest. These findings suggest that treating iron deficiency in patients with HF significantly reduces cardiovascular events and also suggests further investigation of optimal dosing of i.v. iron.

## Abstract (web summary)

This systematic review and meta-analysis provides an updated assessment of the efficacy and safety of intravenous iron therapy in patients with heart failure and iron deficiency, incorporating the results of the newly conducted FAIF-HF2 trial.

## Main

Iron deficiency is common in patients with heart failure (HF) and associated with more severe symptoms, impaired quality of life (QoL) and exercise capacity, an increase in hospitalizations, particularly for HF, and a higher mortality^1–4^. Both American and European guidelines recommend intravenous (i.v.) iron therapy to improve symptoms and QoL in patients with HF and a reduced left ventricular ejection fraction (LVEF) (HFrEF) and iron deficiency^5,6^, but uncertainty persists about the effects of i.v. iron on hospitalizations for HF and mortality^7–12^.

Both the AFFIRM-AHF (a randomized, double-blind placebo-controlled trial comparing the effect of intravenous ferric carboxymaltose on hospitalizations and mortality in iron-deficient subjects admitted for acute heart failure) and IRONMAN (effectiveness of intravenous iron treatment versus standard care in patients with heart failure and iron deficiency) trials narrowly missed their primary endpoints of recurrent HF hospitalization and cardiovascular death^7,10^, but statistical significance was observed after applying pre-specified analyses to mitigate the effects of the COVID-19 pandemic. Using a pre-specified 99% confidence interval (CI), the HEART-FID (ferric carboxymaltose in heart failure with iron deficiency) trial did not show a significant effect of i.v. iron on the composite of mortality, recurrent HF hospitalization and 6-min walking distance, although the result was significant using a conventional 95% CI^11^.

Several meta-analyses have previously been reported^13–16^, concluding that i.v. iron probably reduces the risk of HF hospitalizations but has little effect on cardiovascular or all-cause mortality. Some analyses suggest that the benefits of i.v. iron might be greater in those with a transferrin saturation (TSAT) < 20% and that this criterion alone should be used to define iron deficiency^15^. Furthermore, a Bayesian meta-analysis suggested residual uncertainty about the effects of i.v. iron on the composite of recurrent HF hospitalization and cardiovascular mortality^13^.

The most recent trial, FAIR-HF2, provides substantial additional information that may help address these uncertainties^12^. Therefore, we performed an updated systematic review and meta-analysis to estimate the effect of i.v. iron on key clinical outcomes in patients with HF and iron deficiency overall, as well as in key subgroups.

## Results

### Search and study characteristics

**Extended Data Fig. 1:**
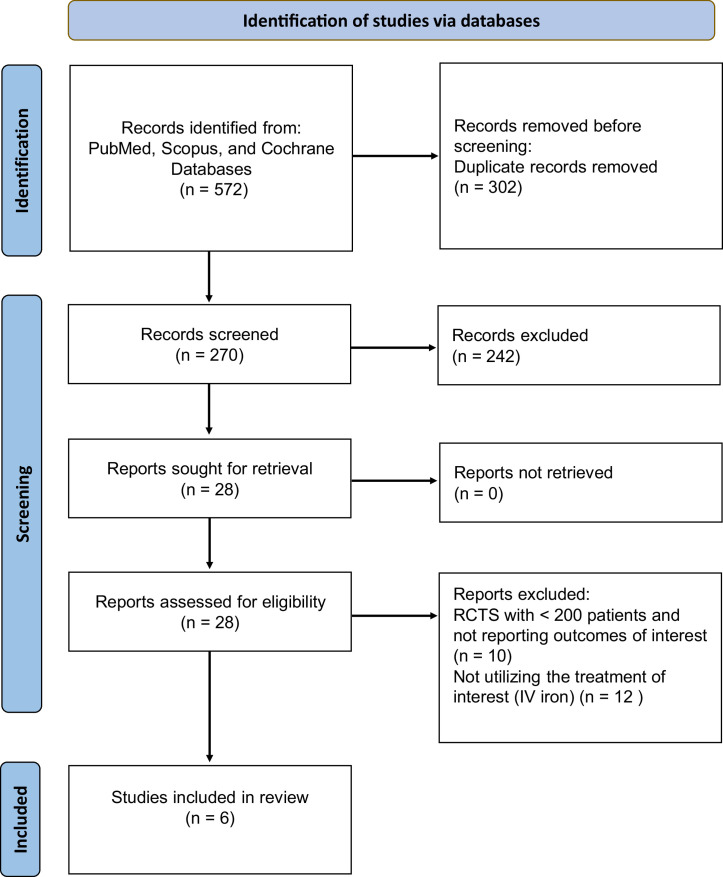
PRISMA flow chart. PRISMA flow chart summarizing study selection process.

The initial search identified 572 potentially relevant articles, but only 6 randomized trials met the inclusion criteria (Extended Data Fig. 1), which had enrolled 7,175 patients, including 3,672 randomized to i.v. iron and 3,503 to control groups. The median duration of follow-up ranged from 6 months to 32 months. The mean or median participant age ranged from 67 years to 74 years and most were men (64%). At baseline, about half the patients were anemic and the percentage with a TSAT < 20% ranged from 40% (Heart-FID) to 83% (AFFIRM-AHF) (Table 1). Five of the six trials used ferric carboxymaltose as the i.v. iron formulation, whereas IRONMAN used ferric derisomaltose. Supplementary Table 1 summarizes the key characteristics of the populations in the included trials.

**Table 1:** Baseline characteristics of the included trials

Trial, year	Sample size (intervention or placebo), follow-up (months)	HF diagnosis	Hemoglobin rang (g dl^−1^)^a^	Women no. (%)	Age (years) mean ± s.d. median (IQR)	LVEF (%) mean ± s.d. median (IQR)	Hemoglobin (g dl^−1^) mean ± s.d. median (IQR)	Ferritin (µg l^−1^) mean ± s.d. median (IQR)	TSAT mean ± s.d. median (IQR)		TSAT < 20% (%)
FAIR-HF2, 2025	1,105 (558 or 547), 17 (8–30)	Chronic HF, LVEF ≤ 45%	9.5–14	368 (33)	70 ± 12	34 ± 8	12.5 ± 1.1	73 ± 55	19 ± 10		68
HEART-FID, 2023	3,065 (1,533 or 1,532), 25 (17–39)	(HF hosp. in last 12 months or ↑NP) NYHA II–IV, LVEF ≤ 40%	>9 and <13.5 for women <15 for men	1,037 (34)	69 ± 11	31 ± 7	12.6 ± 1.4	57 ± 49	24 ± 11	40	
IRONMAN, 2022	1,137 (569 or 568), 32 (22–43)	New or chronic HF NYHA II–IV, LVEF ≤ 40% (current or recent HF hosp. or ↑NP)	>9 and <13 for women <14 for men	300 (26)	74 (67–79)	35 (26–38)	12.1 (11.2–12.8)	50 (30.86)	15 (10–20)	74	
AFFIRM-AHF, 2020	1,108 (558 or 550), 12	Current HF hosp., LVEF < 50%	8–15	494 (42)	71 ± 11	33 ± 10	12.2 ± 1.6	86 ± 65	15 ± 8	83	
CONFIRM-HF, 2015	301 (150 or 151), 12	Chronic HF NYHA II–III, LVEF ≤ 45%, ↑NP	8–15	141 (47)	70 ± 9	37 ± 7	12.4 ± 1.4	57 ± 45	19 ± 13	63	
FAIR-HF, 2009	459 (304/155), 6	Chronic HF NYHA II with LVEF ≤ 40% or NYHA III with LVEF ≤ 45%	9.5–13.5	244 (53)	67 ± 11	33 ± 6	11.9 ± 1.4	56 ± 61	17 ± 11	69	

hosp., hospitalization; NP: natriuretic peptides. Data are reported as mean (s.d.) or median (interquartile range (IQR)). ^a^Hemoglobin: all trials used a definition of iron deficiency of serum ferritin <100 µg l^−1^ or TSAT < 20%. All trials except IROMAN excluded patients with a serum ferritin ≥300 µg l^−1^; IROMAN excluded those with a serum ferritin >400 µg l^−1^.

All trials in this meta-analysis were multicenter and randomized. Although five trials were double blind, IRONMAN was open label; however, its primary endpoint (HF hospitalization and cardiovascular death) was assessed through blinded outcome adjudication to minimize bias. The included randomized controlled trials demonstrated good methodological quality with a low risk of bias in key domains sich as randomization, allocation concealment and outcome assessment. Supplementary Fig. 1 provides an overview of the quality assessment across the included studies.

### Primary endpoint

**Fig. 1:**
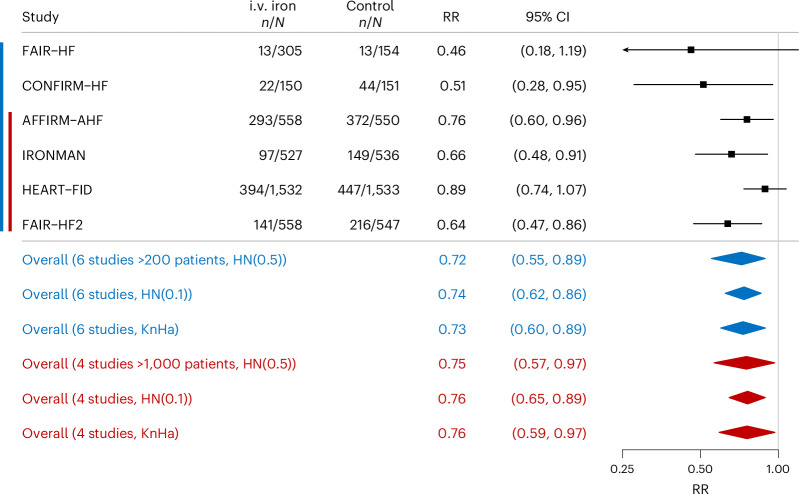
The effect of i.v. iron on the composite endpoint of total (first and recurrent) HF hospitalizations and cardiovascular mortality for the first 12 months of follow-up (*P*_B_ = 0.007, *I*^2^ = 47%). The Forest plot illustrates the impact of i.v. iron on the composite endpoint of total (first and recurrent) HF hospitalizations and cardiovascular mortality during the first 12 months of follow-up using a Bayesian random-effects meta-analysis. Data are presented as RRs with 95% CIs. Sensitivity analyses were conducted by omitting the FAIR-HF and CONFIRM-HF studies, through the application of alternative HN priors and using the Knapp–Hartung (KnHa) approach to random-effects meta-analysis. *n* or *N*, no. of events or no. of participants in the group, respectively. The blue color indicates analysis using six trials and the red color analysis using four trials.

**Fig. 2:**
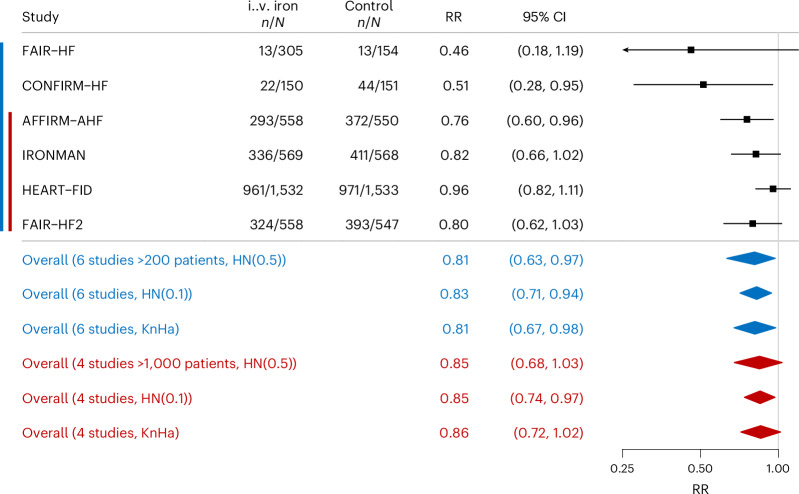
The effect of i.v. iron on the composite endpoint of total (first and recurrent) HF hospitalizations and cardiovascular mortality over the complete length of follow-up (*P*_B_ = 0.022, *I*^2^ = 46%). The Forest plot illustrates the impact of i.v. iron on the composite endpoint of total (first and recurrent) HF hospitalizations and cardiovascular mortality over the complete length of follow-up using a Bayesian random-effects meta-analysis. The data are presented as RRs with 95% CIs. Sensitivity analyses were conducted by omitting the FAIR-HF and CONFIRM-HF studies, through the application of alternative HN priors and using the KnHa approach to random-effects meta-analysis. The blue color indicates analysis using six trials and the red color analysis using four trials.

Compared with patients assigned to control groups, those assigned to i.v. iron had significantly lower rates for the primary composite endpoint by 12 months (RR = 0.72 (95% CI = 0.55–0.89), (posterior) tail probability *(P*_B_)= 0.007, *I*^2^ = 47%; Fig. 1) and at the complete length of follow-up (RR = 0.81 (95% CI = 0.63–0.97), *P*_B_ = 0.022, *I*^2^ = 46%; Fig. 2). Sensitivity analysis using the Knapp–Hartung method yielded similar results for both 12 months (RR = 0.73 (95% CI = 0.60–0.89), *P* = 0.010) and the complete length of follow-up (RR = 0.81 (95% CI = 0.67–0.98), *P* = 0.035).

### Key secondary endpoints

#### Recurrent HF hospitalizations

**Fig. 3:**
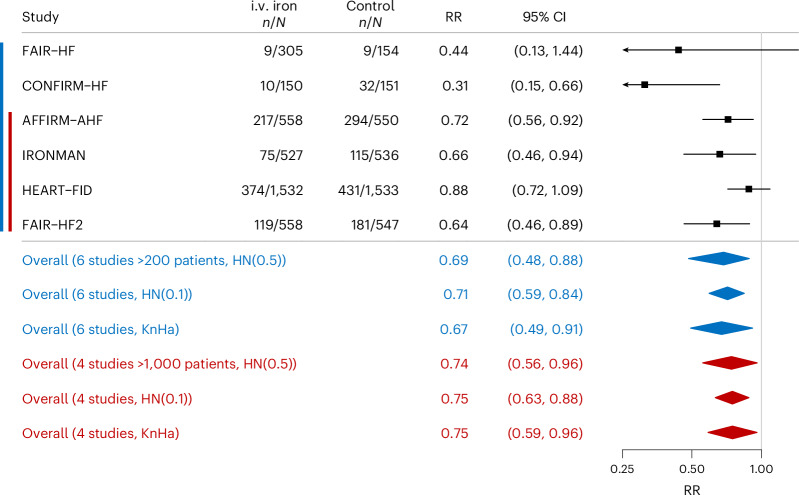
The effect of i.v. iron on recurrent HF hospitalizations for the first 12 months of follow-up (*P*_B_ = 0.009, *I*^2^ = 56%). The Forest plot illustrates the impact of i.v. iron on recurrent HF hospitalizations during the first 12 months of follow-up using a Bayesian random-effects meta-analysis. Data are presented as RRs with 95% CIs. Sensitivity analyses were conducted by omitting the FAIR-HF and CONFIRM-HF studies, through the application of alternative HN priors and using the KnHa approach to random-effects meta-analysis. The blue color indicates analysis using six trials and the red color analysis using four trials.

**Fig. 4:**
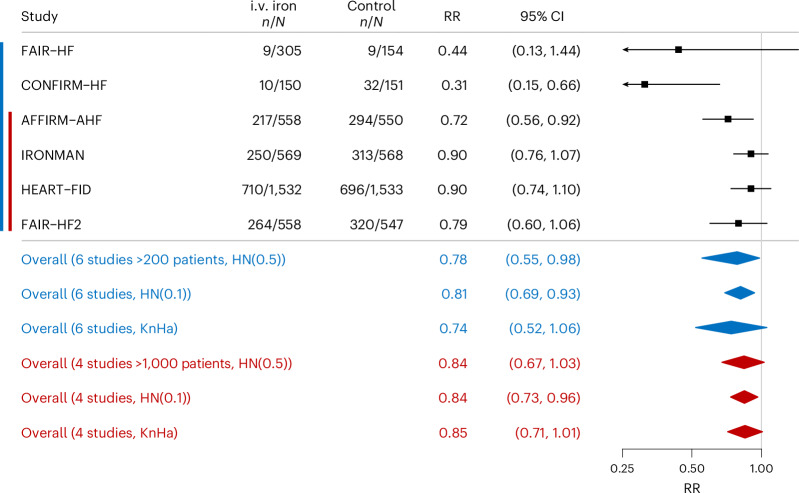
The effect of i.v. iron on recurrent HF hospitalizations over the complete length of follow-up (*P*_B_ = 0.028, *I*^2^ = 59%). The Forest plot shows the effect of i.v. iron on recurrent HF hospitalizations over the complete length of follow-up using a Bayesian random-effects meta-analysis. Data are presented as RRs with 95% CIs. Sensitivity analyses were conducted by omitting the FAIR-HF and CONFIRM-HF studies, through the application of alternative HN priors and using the KnHa approach to random-effects meta-analysis. The blue color indicates analysis using six trials and the red color analysis using four trials.

Compared with patients assigned to control groups, those assigned to i.v. iron had significantly lower rates for recurrent HF hospitalizations by 12 months (RR = 0.69 (95% CI = 0.48–0.88), *P*_B_ = 0.009, *I*^2^ = 56%; Fig. 3) and at the complete length of follow-up (RR = 0.78 (95% CI = 0.55–0.98), *P*_B_ = 0.028, *I*^2^ = 59%; Fig. 4). Sensitivity analysis using the Knapp–Hartung method yielded similar results for both 12 months (RR = 0.67 (95% CI = 0.49–0.91), *P* = 0.021) and complete length of follow-up (RR = 0.74 (95% CI = 0.52–1.06), *P* = 0.081).

#### All-cause and cardiovascular mortality

**Extended Data Fig. 2:**
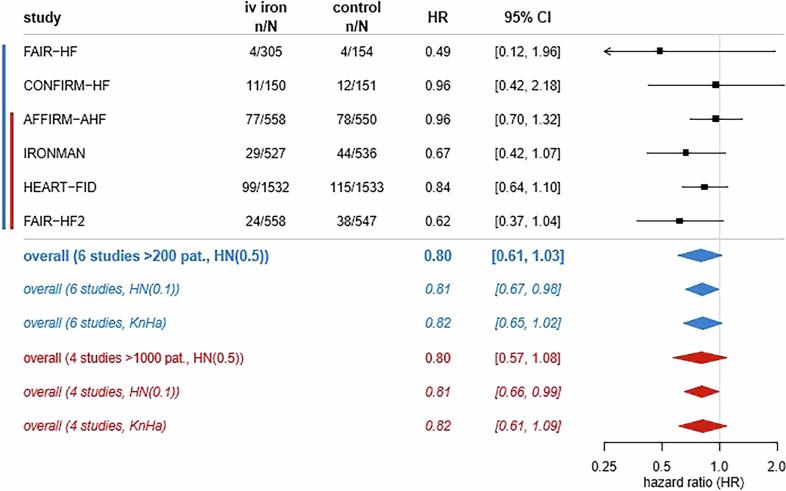
The effect of intravenous iron on cardiovascular mortality for the first 12 months of follow-up (*p*_B_ = 0.071, I^2^ = 24%). The forest plot illustrates the impact of IV iron on cardiovascular mortality during the first 12 months of follow-up using a Bayesian random-effects meta-analysis. Data are presented as hazard ratios (RRs) with 95% credible intervals (CIs). Sensitivity analyses were conducted by omitting the FAIR-HF and CONFIRM-HF studies and by applying alternative half-normal (HN) priors. n / N: number of events / number of subjects in group.

**Extended Data Fig. 3:**
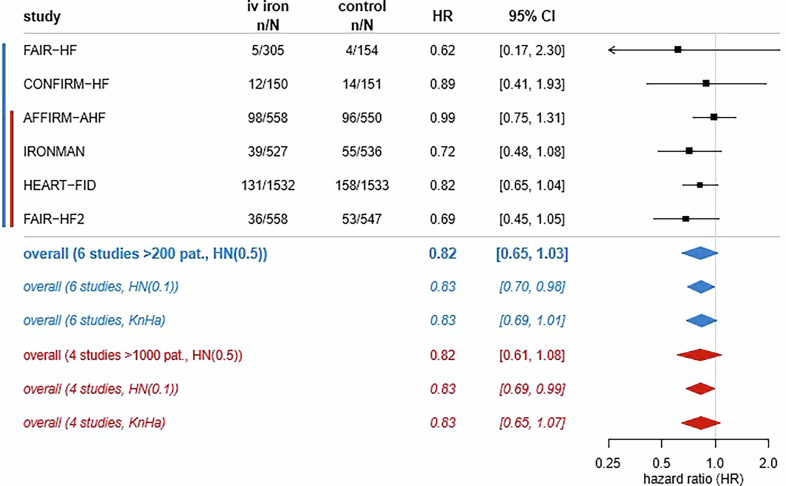
The effect of intravenous iron on all-cause mortality for the first 12 months of follow-up (*p*_B_ = 0.073, I^2^ = 25%). The forest plot shows the impact of IV iron on all-cause mortality during the first 12 months of follow-up using a Bayesian random-effects meta-analysis. Data are presented as hazard ratios (RRs) with 95% credible intervals (CIs). Sensitivity analyses were conducted by omitting the FAIR-HF and CONFIRM-HF studies and by applying alternative half-normal (HN) priors. n / N: number of events / number of subjects in group.

**Extended Data Fig. 4:**
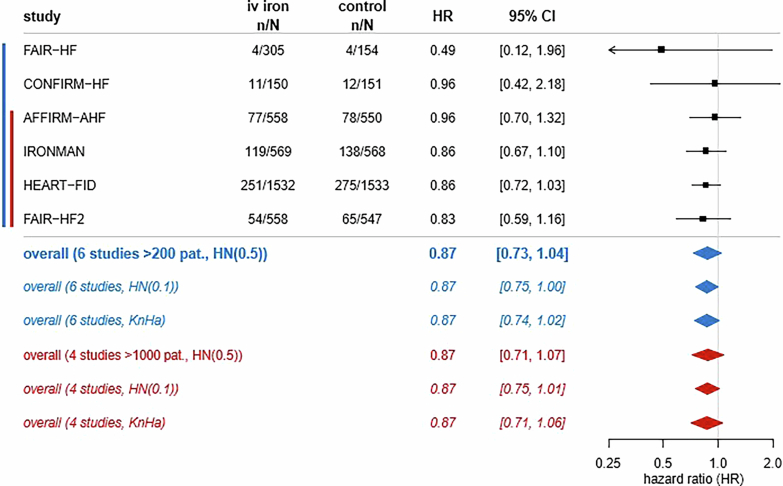
The effect of intravenous iron on cardiovascular mortality over the complete length of follow-up (*p*_B_ = 0.096, I^2^ = 16%). The forest plot illustrates the effect of IV iron on cardiovascular mortality over the complete length of follow-up using a Bayesian random-effects meta-analysis. Data are presented as hazard ratios (HRs) with 95% credible intervals (CIs). Sensitivity analyses were conducted by omitting the FAIR-HF and CONFIRM-HF studies and by applying alternative half-normal (HN) priors. n / N: number of events / number of subjects in group.

**Extended Data Fig. 5:**
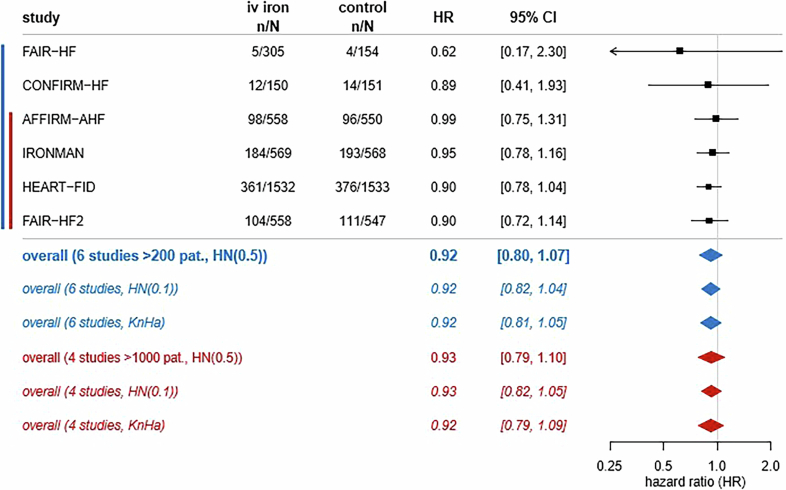
The effect of intravenous iron on all-cause mortality over the complete length of follow-up (*p*_B_ = 0.221, I^2^ = 16%). The forest plot illustrates the effect of IV iron on all-cause mortality over the complete length of follow-up using a Bayesian random-effects meta-analysis. Data are presented as hazard ratios (HRs) with 95% credible intervals (CIs). Sensitivity analyses were conducted by omitting the FAIR-HF and CONFIRM-HF studies and by applying alternative half-normal (HN) priors. n / N: number of events / number of subjects in group.

By 12 months, compared with patients assigned to control groups, those assigned to i.v. iron tended to have lower rates for cardiovascular (HR = 0.80 (95% CI = 0.61–1.03), *P*_B_ = 0.071, *I*^2^ = 24%; Extended Data Fig. 2) and all-cause (HR = 0.82 (95% CI = 0.65–1.03), *P*_B_ = 0.073, *I*^2^ = 25%; Extended Data Fig. 3) mortality. Sensitivity analysis using the Knapp–Hartung method yielded similar results (HR = 0.82 (95% CI = 0.65–1.02), *P* = 0.068) and (HR = 0.83 (95% CI = 0.69–1.01), *P* = 0.060), respectively. At the complete length of follow-up, these trends were attenuated (HR = 0.87 (95% = CI 0.73–1.04), *P*_B_ = 0.096. *I*^2^ = 16%; Extended Data Fig. 4) and (HR = 0.92 (95% CI = 0.80–1.07), *P*_B_ = 0.221, *I*^2^ = 16%; Extended Data Fig. 5), respectively. Sensitivity analysis using the Knapp–Hartung method again yielded similar results (HR = 0.87 (95% = CI 0.74–1.02), *P* = 0.070) and (HR = 0.92 (95% CI = 0.81–1.05), *P* = 0.162), respectively.

### Safety endpoints

Those randomized to i.v. iron or a control group had a similar incidence of infection (odds ratio (OR) = 1.02 (95% CI = 0.66–1.59)) and serious adverse events (OR = 0.91 (95% CI = 0.70–1.15)) (Supplementary Figs. 2 and 3).

### Subgroup analysis

No statistically significant treatment interactions were observed for the primary composite endpoint when patients were stratified according to age, ischemic versus nonischemic etiology, New York Heart Association (NYHA) class, estimated glomerular filtration rate (eGFR), hemoglobin (Hb), ferritin or TSAT (Table 2 and Supplementary Figs. 4–11). However, men appeared to obtain greater benefit than women (Supplementary Fig. 12). There was a significant interaction for sex (ratio of RRs (RRR) 1.40 [95% CI 1.05–1.86]; *P*_B_ = 0.025, *I*^2^ = 23%), with women on average showing no benefit (RR = 0.98 (95% CI = 0.75–1.26)).

**Table 2:** Summary table of subgroup analysis (considering the complete length of follow-up)

Subgroups	Effects in subgroups		Interaction
	RR (95% CI)	RR (95% CI)	RRR (95% CI)
Sex: female/male	0.98 (0.75–1.26)	0.76 (0.56–0.95)	1.40 (1.05–1.86)
Age (years): <69.4 versus ≥69.4	0.73 (0.49, 0.98)	0.87 (0.70, 1.06)	0.84 (0.59, 1.16)
Etiology IHD, Yes/No	0.74 (0.56, 0.92)	0.90 (0.65, 1.18)	0.84 (0.59, 1.22)
TSAT: <20% versus ≥20%	0.77 (0.60, 0.94)	0.96 (0.72, 1.26)	0.85 (0.61, 1.16)
eGFR CKD-EPI (ml per min per 1.73 m^2^): ≤60 versus >60	0.81 (0.65, 0.98)	0.84 (0.60, 1.12)	0.96 (0.70, 1.32)
Hemoglobin (g per dl): <11.8 versus ≥11.8	0.78 (0.58, 1.01)	0.84 (0.62, 1.08)	0.94 (0.62, 1.43)
Ferritin (µg per l): <35 versus ≥35	0.85 (0.65, 1.16)	0.77 (0.53, 1.01)	1.14 (0.74, 1.95)
NYHA class II versus III + IV^a^	0.73 (0.50, 1.02)	0.86 (0.66, 1.09)	0.87 (0.57, 1.29)

^a^In FAIR-HF, there was only 1 event in 82 patients with NYHA class II. Therefore, this subgroup analysis of FAIR-HF was omitted from the meta-analysis. CKD-EPI, Chronic Kidney Disease Epidemiology Collaboration; IHD, ischemic etiology—unknown etiology is included in the no IHD group.

### Sensitivity analyses

Extended Data Tables 2 and 3 contrast the results based on the half-normal (HN) prior (with scale 0.5), with more optimistic (scale 0.1) and more conservative (scale 1.0) alternatives. The results were largely similar with all the three different priors used. Extended Data Tables 4 and 5 show the results of the primary endpoint and key secondary endpoints after exclusion of FAIR-HF and CONFIRM-HF trials, respectively. The results remained largely similar after exclusion of FAIR-HF and CONFIRM-HF trials. The sensitivity analyses using primary endpoint of time to first event for cardiovascular mortality and HF hospitalization are shown in Supplementary Figs. 13 and 14.

## Discussion

This meta-analysis, comprising >7,000 patients, suggests that i.v. iron reduces the composite endpoint of total (first and recurrent) HF hospitalizations and cardiovascular mortality in patients with HF, LVEF < 50% and iron deficiency. The event rate reduction was 28% at 12 months and 19% for all available follow-ups. Both components of the primary endpoint contributed to these outcomes. The directionally positive results for all-cause mortality at 12 months and for all follow-ups document overall safety of i.v. iron therapy in patients with HF.

Benefit was observed most clearly during the first year of follow-up, a finding that may be explained, at least in part, by disruptions caused by the COVID-19 pandemic. In addition, we speculated that this is the result of the impact of higher doses of i.v. iron causing complete correction of iron deficiency (early in each trial). At later times in the trials (that is, after >12 months of follow-up, which was performed in IRONMAN, HEART-FID and FAIR-HF2) doses of i.v. iron were substantially less and adherence to therapy tended to be much less than intended. The average doses of i.v. iron in the first 12 months was approximately 2,000 mg, whereas, in years 2 and 3 of these trials, it was only 300–900 mg per year. It is important to highlight that the initial dose of i.v. iron varied across trials. For most trials, most of the i.v. iron was administered during the first 4–6 weeks, with few patients receiving doses thereafter (Extended Data Table 1). This might also explain the higher treatment effect observed with i.v. iron during the first year after randomization. Maintaining iron repletion throughout follow-up by further doses of i.v. iron, which was often impossible during COVID lockdown periods, might have prevented the attenuation of longer-term benefits of i.v. iron^14,17^. This deserves further exploration in future clinical trials.

Subgroup analyses, with the exception of sex, revealed no significant differences in the effect of i.v. iron on the primary endpoint, including those with a baseline TSAT < 20%. The effect of i.v. iron appeared to be greater in men. Compared with previous meta-analyses, an additional substantial trial (FAIR-HF2, with 1,105 patients) was included. Furthermore, individual-patient data were available from five trials, enabling application of identical subgroup definitions; it also allowed for harmonized analysis methods using the approach of the IRONMAN trial as an example and then applying both Bayesian and conservative frequentist approaches to determine treatment effects overall and in subgroups. It is, therefore, more robust than previous meta-analyses^13–16^.

Several trials have shown that i.v. iron improves symptoms and QoL in patients with HFrEF and iron deficiency^9,18^. This meta-analysis provides evidence that these improvements in well-being, which are of paramount importance to patients, are reflected in a reduction in HF hospitalizations, which not only cause substantial distress for patients but also place significant financial and logistical burdens on healthcare systems^19^.

Some reports have suggested that a TSAT < 20%, rather than serum ferritin, may be a better way to define iron deficiency and identify patients who derive greater benefit from i.v. iron^11,20,21^. Indeed, higher serum ferritin concentrations are associated with worse outcomes in patients with HF, probably because serum ferritin increases with inflammation, which may disguise the presence of iron deficiency^3^. It remains uncertain which blood tests most accurately reflect iron deficiency. Indeed, it is likely that some patients without iron deficiency were included in the trials and this may have diluted the benefits of i.v. iron supplementation. Importantly, FAIR-HF2 was the first trial to pre-specify an analysis of the effects of i.v. iron on the subgroup of patients with a TSAT < 20% as part of its primary outcome. Although patients with a TSAT < 20% had, overall, a higher rate of events, the effect of i.v. iron in relative terms was similar for those with a TSAT above or below 20%. This meta-analysis also failed to show a statistically significant interaction between the effects of i.v. iron on the primary outcome and TSAT. However, patients with a TSAT < 20% did have a worse prognosis and so, even if the relative benefits for those with a TSAT above or below 20% are similar, the absolute benefit will be greater for patients with a TSAT < 20%. These results help inform the debate on whether TSAT < 20% should be the sole criterion for identifying iron deficiency in patients with HF.

Previous analyses have suggested that patients with a nonischemic cause for HF might not benefit from i.v. iron^22^. We could not confirm this, nor did we observe any difference in effects based on age, NYHA class, eGFR, hemoglobin or ferritin. It is interesting that we did observe a statistically significant subgroup interaction according to sex, with women possibly deriving less benefit. This might be a chance finding or confounded by differences in patient characteristics such as age, underlying ischemic heart disease (IHD) or iron deficiency markers, and was not observed consistently across trials.

There are also concerns about the safety of giving high amounts of i.v. iron, but the recent FAIR-HF2 trial found that higher cumulative dosing with i.v. iron was safe and well tolerated. The current meta-analysis identified no safety concerns with infections or other serious adverse events, for which rates were similar in the control and i.v. iron groups. This is in contrast to reports from the IRONMAN trial, which showed trends toward fewer infection-related events with i.v. ferric derisomaltose (a pre-specified endpoint)^23,24^.

Some limitations to this analysis should be considered. The absence of individual participant data from the IRONMAN trial limited the ability to adjust for covariates for additional subgroup analyses. Also, there was heterogeneity among trials in terms of i.v. iron formulations, dose used, whether patients were enrolled in or out of hospital and national differences in characteristics or healthcare services that might affect hospitalization rates. However, showing similar effects across diverse populations might also be considered a strength of this analysis. Last, two of the six trials included (FAIR-HF and CONFIRM) did not have a clinical outcome as the primary endpoint and the findings from these trials may be uncertain, given the wide CIs.

In conclusion, the totality of evidence suggests that treating iron deficiency in patients with HFrEF with i.v. iron significantly reduces the composite outcome of recurrent HF hospitalizations and cardiovascular mortality, which reflected reductions in both components, but particularly HF hospitalizations. Treatment effects were greatest in the first year after randomization and consistent across various subgroups, including baseline TSAT. Further research is needed to confirm whether women obtain less benefit from i.v. iron and, if so, why.

## Methods

This meta-analysis adheres to Preferred Reporting Items Systematic Reviews and Meta-Analyses (PRISMA) recommendations^25^. Ethical committee approval was not required because all analyses were based on existing data. The protocol was registered in PROSPERO before data extraction and analysis (registration no. CRD42025635165).

### Data sources and search strategy

A comprehensive search of MEDLINE and Scopus was conducted, without language restrictions, from the inception of these databases through the first week of January 2025, by two independent investigators (M.S.K. and K.M.T.). The detailed search strategy is provided in Supplementary Table 2. To ensure that no relevant publications were overlooked, the search was supplemented with a review of ClinicalTrials.gov and references in recent reviews and meta-analyses. All retrieved articles were imported into Endnote X7 (Clarivate Analytics) to identify and remove duplicates. Titles and abstracts were initially screened, followed by a full-text review to confirm eligibility. The two independent reviewers (M.S.K. and K.M.T.) evaluated the studies, with any disagreements resolved through discussion with a third reviewer (S.D.A.).

### Inclusion criteria

Randomized trials comparing i.v. iron with placebo or standard or usual care in adults with HF, iron deficiency and a left ventricular ejection fraction (LVEF) ≤50% reporting HF hospitalizations and mortality that enrolled ≥200 patients and lasted ≥24 weeks were included.

### Data extraction and risk-of-bias assessment

Relevant data were extracted into an Excel spreadsheet. Risk of bias was evaluated by two authors (M.S.K. and K.M.T.) using the Cochrane risk-of-bias tool^26^, focusing on random sequence generation, allocation concealment, blinding of participants or personnel and outcomes, completeness of outcome data and selective reporting. Each trial was classified as having a low, high or unclear risk of bias for each domain.

### Outcomes and subgroups

The primary endpoint was the composite of recurrent HF hospitalizations (total events) or cardiovascular death (1) within 12 months of randomization and (2) during the entire follow-up. A composite of recurrent HF hospitalizations or cardiovascular mortality was chosen as the primary endpoint because it was the primary endpoint for AFFIRM-AHF, IRONMAN and FAIR-HF2, a key secondary endpoint in HEART-FID, as well as for several previous meta-analyses. It is also worth noting that the same or similar primary endpoint has been used in many other large HF trials that have shaped international practice and guidelines. Key secondary endpoints for this analysis included total HF hospitalizations, cardiovascular mortality and all-cause mortality (1) within 12 months of randomization and (2) during the entire follow-up period. Safety endpoints included serious adverse events or hospitalizations resulting from infections within 12 months of randomization and over the entire follow-up period.

We obtained patient-level data for FAIR-HF, FAIR-HF2, CONFIRM-HF, AFFIRM-AHF and HEART-FID trials and applied the analysis methods and subgroup definitions of the IRONMAN trial, for which only trial-level data are available at this time. The subgroup analyses for the primary endpoint focused on sex, age (<69.4 versus ≥69.4 years), etiology of HF (ischemic versus nonischemic), TSAT (<20% versus ≥20%), eGFR (calculated using the Chronic Kidney Disease Epidemiology Collaboration equation, ≤60 versus >60 ml min^−1^ 1.73 m^−2^), hemoglobin (<11.8 versus ≥11.8 g dl^−1^), ferritin (<35 versus ≥35 μg l^−1^) and NYHA class (II versus III + IV). The cut-offs for these subgroups were taken from the IRONMAN subgroup analyses. Outcomes at 12 months for the IRONMAN trial were extracted from the IRONMAN publication’s Supplementary Material Table S3 (ref. ^7^).

### Statistical analysis

Using the same statistical methods as the IRONMAN trial, FAIR-HF, CONFIRM-HF, AFFIRM-AHF, HEART-FID and FAIR-HF2 trials were re-analyzed using the Lin–Wei–Yang–Ying model for (1) the composite outcome of total (first and recurrent) HF hospitalizations and cardiovascular death and (2) total HF hospitalizations alone^27^. The primary analyses in IRONMAN had been adjusted for recruitment context (hospital admission or outpatient). As all the other trials were conducted in either of these contexts, the analyses were not adjusted for recruitment context. However, re-analyses were adjusted for region because they were conducted internationally, whereas IRONMAN was conducted in the United Kingdom only. Time-to-event analyses utilized Cox’s proportional hazards regressions extracted from the publications.

Random-effects meta-analyses were conducted on aggregated data using the normal–normal hierarchical model (NNHM) within a Bayesian framework^28^. This approach, in contrast to frequentist meta-analysis, treats both data and model parameters as random variables, incorporates prior distributions, accounts for uncertainty in estimating between-trial heterogeneity and allows sensitivity analyses by adjusting distributional assumptions and incorporating prior knowledge. Measures of effect included HRs for time-to-event outcomes (for example, cardiovascular and all-cause mortality) and RRs for recurrent events (for example, total HF hospitalizations with or without cardiovascular death). A weakly informative prior for between-trial heterogeneity (*τ*), specifically an HN prior with a scale of 0.5, was applied^29^, whereas uninformative priors were used for treatment and interaction effects. Sensitivity analyses using alternative priors such as 0.1 and 1.0 were also conducted to further strengthen the methodological rigor. We also conducted a sensitivity analysis by excluding FAIR-HF and CONFIRM-HF trials because they were relatively smaller trials focusing on exercise capacity and symptoms. Results were summarized by marginal posterior medians of the log(RR), log(HR), log(RRR) and the between-trial heterogeneity *τ*. Between-trial heterogeneity was visualized in Forest plots. Bayesian meta-analyses were conducted using the R package bayesmeta^30^. As supporting analyses, frequentist analyses were also performed, using the Knapp–Hartung approach to random-effects meta-analysis with the Paule–Mandel estimator for the between-trial heterogeneity^31,32^. *P* values were reported for the frequentist meta-analyses. The closest equivalent to *P* values that one may compute from a Bayesian analysis is the corresponding *P*_B_ which was reported. All analyses were performed using R (v.4.4 or higher) or SAS (v.9.4 or higher).

### Reporting summary

Further information on research design is available in the Nature Portfolio Reporting Summary linked to this article.

## Online content

Any methods, additional references, Nature Portfolio reporting summaries, source data, extended data, supplementary information, acknowledgements, peer review information; details of author contributions and competing interests; and statements of data and code availability are available at 10.1038/s41591-025-03671-1.

## Supplementary information

Supplementary Information Supplementary Tables 1 and 2 and Figs. 1–14.

Reporting Summary

## Figures and Tables

**Extended Data Table 1:**
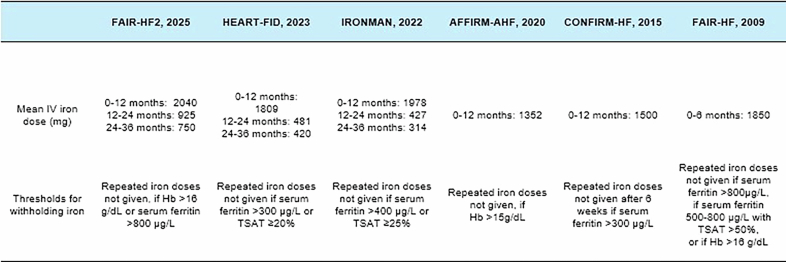
Overview of i.v. iron dosing

This table presents the mean i.v. iron dose and thresholds for withholding iron across all included trials.

**Extended Data Table 2:**
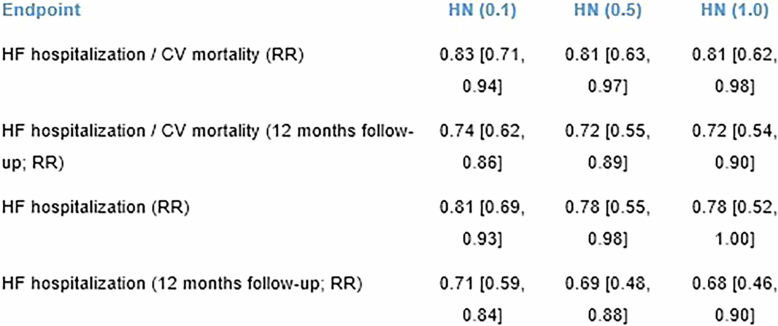
Effect estimates based on sensitivity analyses of the original results

Instead of an HN prior with scale 0.5, scales of 0.1 and 1.0 are used. This table presents effect estimates based on sensitivity analyses of the original results. Instead of the default HN prior with a scale of 0.5, alternative priors with scales of 0.1 (HN(0.1)) and 1.0 (HN(1.0)) were applied.

**Extended Data Table 3:**
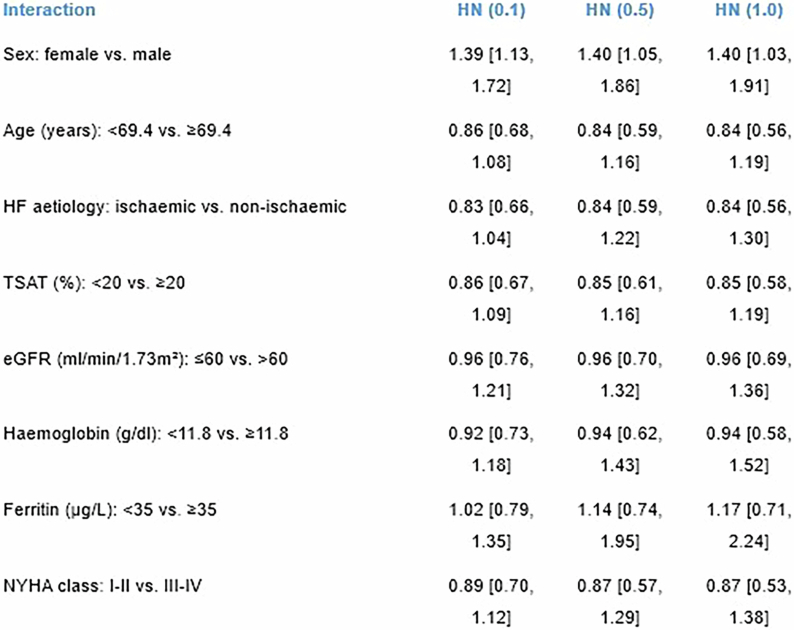
Interaction estimates (RRRs) based on sensitivity analyses of the original results from Table 2

Instead of a HN prior with scale 0.5, scales of 0.1 and 1.0 are used. This table presents RRRs based on sensitivity analyses of the original results from Table 2. Instead of the default HN prior with a scale of 0.5, alternative priors with scales of 0.1 (HN(0.1)) and 1.0 (HN(1.0)) were applied.

**Extended Data Table 4:**
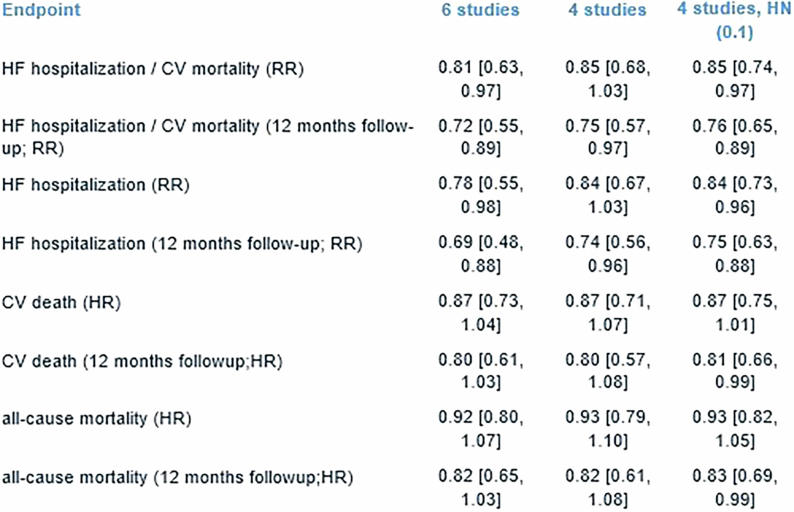
Effect estimates based on sensitivity analyses of the original results

Instead of using the full set of six studies, the FAIR-HF and CONFIRM-HF studies were omitted. In addition, an analysis of the four studies based on a HN(0.1) prior is also shown. This table presents effect estimates based on sensitivity analyses of the original results. Sensitivity analyses were conducted by omitting the FAIR-HF and CONFIRM-HF studies. In addition, an analysis of the remaining four studies using a HN prior with a scale of 0.1 is also shown.

**Extended Data Table 5:**
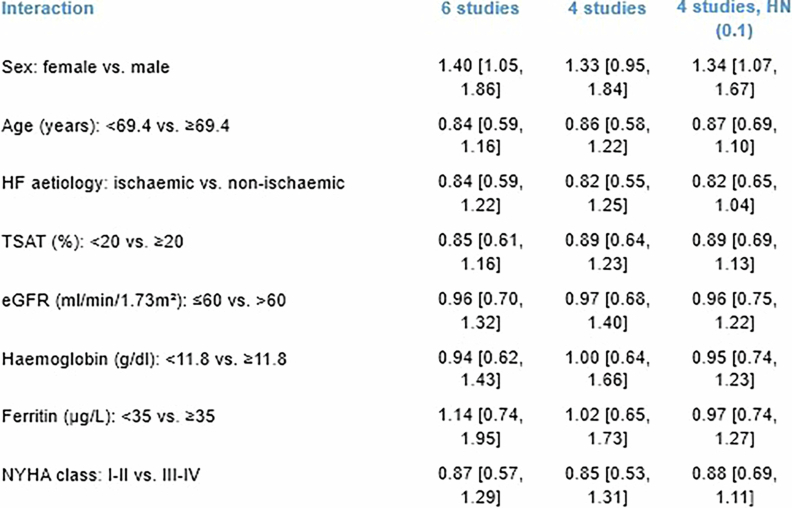
Interaction estimates (RRRs) based on sensitivity analyses of the original results shown in Table 2

Instead of using the full set of six studies, the FAIR-HF and CONFIRM-HF studies were omitted. This table presents RRRs based on sensitivity analyses of the original results shown in Table 2. Sensitivity analyses were conducted by omitting the FAIR-HF and CONFIRM-HF studies.

## Data Availability

Requests for data by any researcher will be considered upon request by contacting the corresponding author. Requests will be processed within an estimated timeframe of 2–4 weeks.
