# Supplementary material for: Systematic review and meta-analysis of intravenous iron therapy for patients with heart failure and iron deficiency
Source: Nat Med. 2025 Mar 30;31(8):2640–6. doi: 10.1038/s41591-025-03671-1 (PMC12353798; doi:10.1038/s41591-025-03671-1)

nature medicine

Supplementary information https://doi.org/10.1038/s41591-025-03671-1

Systematic review and meta-analysis of intravenous iron therapy for patients with heart failure and iron deficiency

In the format provided by the authors and unedited

Supplementary Data

Supplementary Table 1: Key Characteristics of Clinical Trials Evaluating IV Iron Therapy in Patients with HFrEF

Supplementary Table 2: Summarizes the search strategy used on PubMed, Scopus and Cochrane database.

Supplementary Figure 1: Summary of the risk of bias assessment for the included trials.

Supplementary Figure 2: The forest plot illustrates the effect of intravenous iron on infections or infestations for the entire duration of follow-up using Bayesian random-effects meta-analysis. Data are presented as odd ratios (ORs) with 95% credible intervals (CIs). Sensitivity analyses were conducted by omitting the FAIR-HF and CONFIRM-HF studies, by applying alternative half-normal (HN) priors and and by using the Knapp-Hartung (KnHa) approach to random-effects meta-analysis. N: number of subjects in group; pat=patients. Blue color indicates analysis using all available trials while red color indicates analysis excluding FAIR-HF and CONFIRM-HF.

Supplementary Figure 3: The forest plot illustrates the effect of intravenous iron on serious adverse events over the complete length of follow-up for using Bayesian random-effects meta-analysis. . Data are presented as odd ratios (ORs) with 95% credible intervals (CIs). Sensitivity analyses were conducted by omitting the FAIR-HF and CONFIRM-HF studies, by applying alternative half-normal (HN) priors and and by using the Knapp-Hartung (KnHa) approach to random-effects meta-analysis. N: number of subjects in group; pat=patients. Blue color indicates analysis using all available trials while red color indicates analysis excluding FAIR-HF and CONFIRM-HF.

Supplementary Figure 4: The forest plot displays the subgroup analysis of composite endpoint of total (first and recurrent) heart failure hospitalizations and cardiovascular mortality based on sex. Data are presented as ratio of rate ratios (RRRs) with 95% credible intervals (CIs). Sensitivity analyses were conducted by omitting the FAIR-HF and CONFIRM-HF studies, by applying alternative half-normal (HN) priors and and by using the Knapp-Hartung (KnHa) approach to random-effects meta-analysis. N: number of subjects in group; pat=patients. Blue color indicates

analysis using all available trials while red color indicates analysis excluding FAIR-HF and CONFIRM-HF.

Supplementary Figure 5: The forest plot illustrates the subgroup analysis of the composite endpoint of total (first and recurrent) heart failure hospitalizations and cardiovascular mortality, stratified by age. Data are presented as ratio of rate ratios (RRRs) with 95% credible intervals (CIs). Sensitivity analyses were conducted by omitting the FAIR-HF and CONFIRM-HF studies, by applying alternative half-normal (HN) priors, and and by using the Knapp-Hartung (KnHa) approach to random-effects meta-analysis. N: number of subjects in group; pat=patients. Blue color indicates analysis using all available trials while red color indicates analysis excluding FAIR-HF and CONFIRM-HF.

Supplementary Figure 6: The forest plot displays the subgroup analysis of the composite endpoint of total (first and recurrent) heart failure hospitalizations and cardiovascular mortality, stratified by heart failure etiology. Data are presented as ratio of rate ratios (RRRs) with 95% credible intervals (CIs). Sensitivity analyses were conducted by omitting the FAIR-HF and CONFIRM-HF studies, by applying alternative half-normal (HN) priors and by using the Knapp-Hartung (KnHa) approach to random-effects meta-analysis. N: number of subjects in group; pat=patients. Blue color indicates analysis using all available trials while red color indicates analysis excluding FAIR-HF and CONFIRM-HF.

Supplementary Figure 7: The forest plot illustrates the subgroup analysis for the composite endpoint of total (first and recurrent) heart failure hospitalizations and cardiovascular mortality stratified by baseline transferrin saturation. Data are presented as ratio of rate ratios (RRRs) with 95% credible intervals (CIs). Sensitivity analyses were conducted by omitting the FAIR-HF and CONFIRM-HF studies, by applying alternative half-normal (HN) priors, and by using the Knapp-Hartung (KnHa) approach to random-effects meta-analysis. N: number of subjects in group; pat=patients. Blue color indicates analysis using all available trials while red color indicates analysis excluding FAIR-HF and CONFIRM-HF.

Supplementary Figure 8: The forest plot illustrates the subgroup analysis for the composite endpoint of total (first and recurrent) heart failure hospitalizations and cardiovascular mortality, based on estimated glomerular filtration rate. Data are presented as ratio of rate ratios (RRRs) with 95% credible intervals (CIs). Sensitivity analyses were conducted by omitting the FAIR-HF and CONFIRM-HF studies, by applying alternative half-normal (HN) priors, and by using the Knapp-Hartung (KnHa) approach to random-effects meta-analysis. N: number of subjects in group; pat=patients. Blue color indicates analysis using all available trials while red color indicates analysis excluding FAIR-HF and CONFIRM-HF.

Supplementary Figure 9: The forest plot illustrates the subgroup analysis composite endpoint of total (first and recurrent) heart failure hospitalizations and cardiovascular mortality based on hemoglobin. Data are presented as ratio of rate ratios (RRRs) with 95% credible intervals (CIs). Sensitivity analyses were conducted by omitting the FAIR-HF and CONFIRM-HF studies, by applying alternative half-normal (HN) priors, and by using the Knapp-Hartung (KnHa) approach to random-effects meta-analysis. N: number of subjects in group; pat=patients. Blue color indicates analysis using all available trials while red color indicates analysis excluding FAIR-HF and CONFIRM-HF.

Supplementary Figure 10: The forest plot illustrates the subgroup analysis of the composite endpoint of total (first and recurrent) heart failure hospitalizations and cardiovascular mortality, stratified by ferritin concentrations. Data are presented as ratio of rate ratios (RRRs) with 95% credible intervals (CIs). Sensitivity analyses were conducted by omitting the FAIR-HF and CONFIRM-HF studies, by applying alternative half-normal (HN) priors, and by using the Knapp-Hartung (KnHa) approach to random-effects meta-analysis. N: number of subjects in group; pat=patients. Blue color indicates analysis using all available trials while red color indicates analysis excluding FAIR-HF and CONFIRM-HF

Supplementary Figure 11: The forest plot illustrates the subgroup analysis of composite endpoint of total (first and recurrent) heart failure hospitalizations and cardiovascular mortality, stratified by NYHA class. Data are presented as ratio of rate ratios (RRRs) with 95% credible intervals (CIs). Sensitivity analyses were conducted by omitting the FAIR-HF and CONFIRM-HF studies, by

applying alternative half-normal (HN) priors, and by using the Knapp-Hartung (KnHa) approach to random-effects meta-analysis. N: number of subjects in group; pat=patients. Blue color indicates analysis using all available trials while red color indicates analysis excluding FAIR-HF and CONFIRM-HF.

Supplementary Figure 12: The forest plot shows the effect of IV iron on the composite endpoint of total (first and recurrent) HF hospitalizations and cardiovascular mortality with respect to men and women. Data are presented as risk ratios (RRs) with 95% credible intervals (CIs).

Supplementary Figure 13: Effect estimates based on sensitivity analyses using the primary endpoint of time to first event for cardiovascular mortality and HF hospitalization, with a half-normal prior of HN (0.5). Data are presented as hazard ratios (HRs) with 95% credible intervals (CIs).

Supplementary Figure 14: Effect estimates based on sensitivity analyses using the primary endpoint of time to first event for cardiovascular mortality and HF hospitalization, with a half-normal prior of HN (0.1). Data are presented as hazard ratios (HRs) with 95% credible intervals (CIs).

Supplementary Table 1: Key Characteristics of Clinical Trials Evaluating IV Iron Therapy in Patients with HFrEF.

Trial, Year	Study design	Included pateint population	Blinding	IV Iron	Primary endpoints
**FAIR-HF2, 2025**	Multicenter, international, placebo-controlled, randomised trial	Patients ≥18 years with chronic HFrEF (≥3 months), LVEF ≤45%, iron deficiency (ferritin <100 ng/mL or 100–299 ng/mL with TSAT <20%), and Hb 9.5–14.0 g/dL. Eligible patients were either re-stabilized for discharge (NYHA II/III) or stable ambulatory with prior HF hospitalization (≤12 months) or elevated natriuretic peptides.	Double-blind study with concealed allocation, black syringes, and blinded outcome assessment.	Ferric carboxymaltose	Time to first CV death or HF hospitalization, total HF hospitalizations, and time to first CV death or HF hospitalization in patients with TSAT <20%
**HEART-FID, 2023**	Multicenter, randomized, placebo-controlled trial	Patients ≥18 years with HF, LVEF ≤40%, Hb >9.0 g/dL but <13.5 g/dL (women) or <15.0 g/dL (men), iron deficiency (ferritin <100 ng/mL or 100–300 ng/mL with TSAT <20%), and either recent HF hospitalization (≤12 months) or elevated natriuretic peptides.	Double-blind study with blinded patients, investigators, and study personnel assessing outcomes.	Ferric carboxymaltose	Composite including death, HF hospitalization, and change in 6-MWT distance
**IRONMAN, 2022**	Multicenter, investigator-initiated, prospective, randomised trial	Patients ≥18 years with symptomatic HF, LVEF ≤45% (past 24 months), iron deficiency (ferritin <100 µg/L or TSAT <20%), recent HF hospitalization (≤6 months) or elevated natriuretic peptides and Hb 9-14g/dL.	Open-label trial (no blinding of patients or staff) due to challenges in masking intravenous iron. However, outcome adjudication was blinded to minimize bias in assessing HF hospitalization and CV death.	Ferric derisomaltose	Composite of HF hospitalization and CV death

**AFFIRM-AHF, 2020**	Multicenter, randomized, placebo controlled trial	Patients ≥18 years, hospitalized for AHF with signs/symptoms, elevated natriuretic peptides, LVEF <50%, iron deficiency (ferritin <100 ng/mL or 100–299 ng/mL with TSAT <20%), and received ≥40 mg IV furosemide (or equivalent).	Double-blind design with unmasked personnel responsible for drug preparation and administration. Blinding was maintained using black syringes and a curtain or similar partition to prevent exposure to patients and site personnel.	Ferric carboxymaltose	Composite of total HF hospitalisations and CV death
**CONFIRM-HF, 2015**	Multicenter, randomized, placebo controlled trial	Patients ≥18 years with stable ambulatory HF (NYHA II/III), LVEF ≤45%, elevated natriuretic peptides (BNP >100 pg/mL or NT-proBNP >400 pg/mL), iron deficiency (ferritin <100 ng/mL or 100–300 ng/mL with TSAT <20%), Hb <15 g/dL, and able to perform the 6MWT.	Double-blind design with unblinded staff handling treatment preparation and administration using black syringes and a partition to maintain blinding.	Ferric carboxymaltose	Change in 6MWT distance
**FAIR-HF, 2009**	Multicenter, randomized, placebo controlled trial	Patients with chronic HF (NYHA class II/III), LVEF ≤40% for NYHA II or ≤45% for NYHA III, Hb 9.5–13.5 g/dL, and iron deficiency (ferritin <100 µg/L or 100–300 µg/L with TSAT <20%).	Double-blind design with unblinded personnel preparing and administering treatment using black syringes and a barrier to maintain patient blinding.	Ferric carboxymaltose	Self-reported Patient Global Assessment and NYHA functional class

Notes: HFrEF: Heart failure with reduced ejection fraction; LVEF: Left ventricular ejection fraction; NYHA: New York Heart Association; HF: Heart failure; TSAT: Transferrin saturation; Hb: Hemoglobin; BNP: B-type natriuretic peptide; NT-proBNP: N-terminal pro-B-type natriuretic peptide; AHF: Acute heart failure; eGFR: Estimated glomerular filtration rate; ACS: Acute coronary syndrome; TIA: Transient ischemic attack; CABG: Coronary artery bypass grafting; PTCA: Percutaneous transluminal coronary angioplasty;CV: Cardiovascular ;CRT: Cardiac resynchronization therapy; 6MWT: Six-minute walk test.

Supplementary Table 2: Search Strategy for PubMed, Scopus and Cochrane

PubMed:<br>(("Heart Failure"[MeSH Terms] OR "heart failure with reduced ejection fraction"[All Fields] OR "HFrEF"[All Fields] OR "chronic heart failure"[All Fields]) AND ("iron deficiency"[All Fields] OR "iron-deficient"[All Fields]) AND ("Intravenous Iron"[All Fields] OR "IV iron therapy"[All Fields] OR "iron supplementation"[All Fields] OR "ferric carboxymaltose"[All Fields] OR "ferric derisomaltose"[All Fields]) AND ("Randomized Controlled Trials as Topic"[MeSH Terms] OR "randomized controlled trial"[All Fields] OR "RCTs"[All Fields] OR "clinical trials"[All Fields] OR "systematic review"[All Fields] OR "meta-analysis"[All Fields])) AND (meta-analysis[Filter])<br><br># of results: 32
Cochrane:<br>((Heart Failure) OR (heart failure with reduced ejection fraction) OR (HFrEF)) AND ((Iron Deficiency) OR (iron deficiency) OR (iron-deficient)) AND ((intravenous iron) OR (IV iron therapy) OR (iron supplementation) OR (ferric carboxymaltose) OR (ferric derisomaltose)) AND ((randomized controlled trial) OR (RCT) OR (clinical trials) OR (meta-analysis))<br><br># of results: 227
Scopus:<br>(TITLE-ABS-KEY (("heart failure" OR "HFrEF" OR "chronic heart failure") AND ("iron deficiency" OR "iron-deficient") AND ("intravenous iron" OR "IV iron therapy" OR "iron supplementation" OR "ferric carboxymaltose" OR "ferric derisomaltose")) AND KEY ("randomized controlled trial" OR "RCTs" OR "clinical trials" OR "systematic review" OR "meta-analysis"))<br><br># of results: 313

Supplementary Figure 1: Risk of Bias Summary

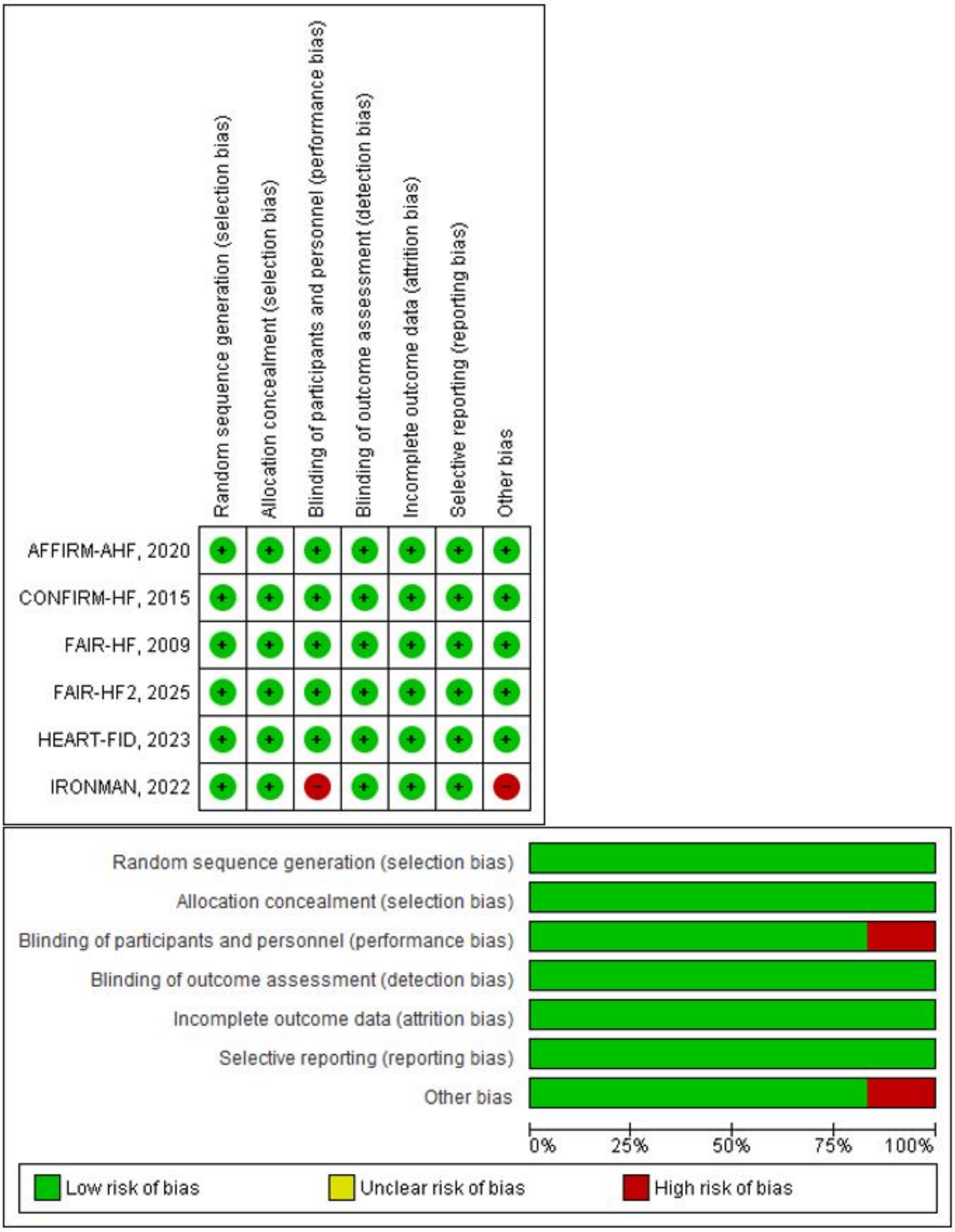

Supplementary Figure 2: The effect of intravenous iron on infections or infestations over the complete length of follow-up using Bayesian random-effects meta-analysis (p_B=0.902).

study	iv iron patients/N	control patients/N	OR	95% CI
FAIR-HF	50 / 305	24 / 154	1.06	[0.62, 1.81]
AFFIRM-AHF	102 / 559	121 / 551	0.79	[0.59, 1.06]
IRONMAN	142 / 559	162 / 568	0.85	[0.66, 1.11]
FAIR-HF2	165 / 558	118 / 547	1.53	[1.16, 2.01]
**overall (4 studies >200 pat., HN(0.5))**			**1.02**	**[0.66, 1.59]**
overall (4 studies, HN(0.1))			*1.02*	*[0.82, 1.28]*
overall (4 studies, KnHa)			*1.02*	*[0.62, 1.67]*
overall (3 studies >1000 pat., HN(0.5))			1.01	[0.56, 1.81]
overall (3 studies, HN(0.1))			*1.02*	*[0.79, 1.30]*
overall (3 studies, KnHa)			*1.01*	*[0.42, 2.47]*

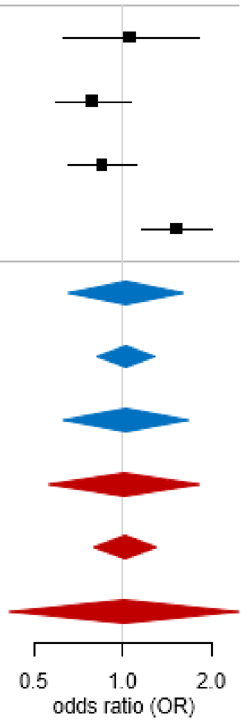

Supplementary Figure 3: The effect of intravenous iron on serious adverse events complete length of follow-up using Bayesian random-effects meta-analysis (p_B=0.347).

study	iv iron patients/N	control patients/N	OR	95% CI
CONFIRM−HF	43 / 152	53 / 152	0.74	[0.45, 1.20]
AFFIRM−AHF	250 / 559	282 / 551	0.77	[0.61, 0.98]
IRONMAN	410 / 559	435 / 568	0.84	[0.64, 1.10]
HEART−FID	581 / 1532	537 / 1533	1.13	[0.98, 1.31]
FAIR−HF2	269 / 558	273 / 547	0.93	[0.74, 1.18]
**overall (5 studies >200 pat., HN(0.5))**			**0.91**	**[0.70, 1.15]**
overall (5 studies, HN(0.1))			*0.93*	*[0.78, 1.08]*
overall (5 studies, KnHa)			*0.91*	*[0.73, 1.14]*
overall (4 studies >1000 pat., HN(0.5))			0.93	[0.68, 1.24]
overall (4 studies, HN(0.1))			*0.94*	*[0.79, 1.10]*
overall (4 studies, KnHa)			*0.93*	*[0.70, 1.23]*

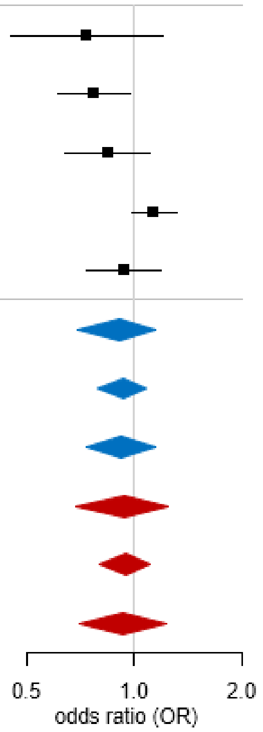

Supplementary Figure 4: Analysis of interaction between sex and iv iron effects on the composite endpoint of total (first and recurrent) heart failure hospitalizations and cardiovascular mortality. Shown are the ratios of rate ratios (RRRs) comparing the treatment effects in each study's two subgroups (women vs. men) (p_B=0.025, I^2=23%).

study	N	RRR	95% CI
FAIR−HF	459	1.48	[0.41, 5.34]
CONFIRM−HF	301	2.23	[1.04, 4.77]
AFFIRM−AHF	1108	1.56	[1.15, 2.13]
IRONMAN	1137	1.06	[0.62, 1.82]
HEART−FID	3065	1.23	[0.88, 1.71]
FAIR−HF2	1105	1.40	[0.73, 2.69]
**overall (6 studies >200 pat., HN(0.5))**		**1.40**	**[1.05, 1.86]**
overall (6 studies, HN(0.1))		*1.39*	*[1.13, 1.72]*
overall (6 studies, KnHa)		*1.39*	*[1.08, 1.78]*
overall (4 studies >1000 pat., HN(0.5))		1.33	[0.95, 1.84]
overall (4 studies, HN(0.1))		*1.34*	*[1.07, 1.67]*
overall (4 studies, KnHa)		*1.35*	*[0.98, 1.86]*

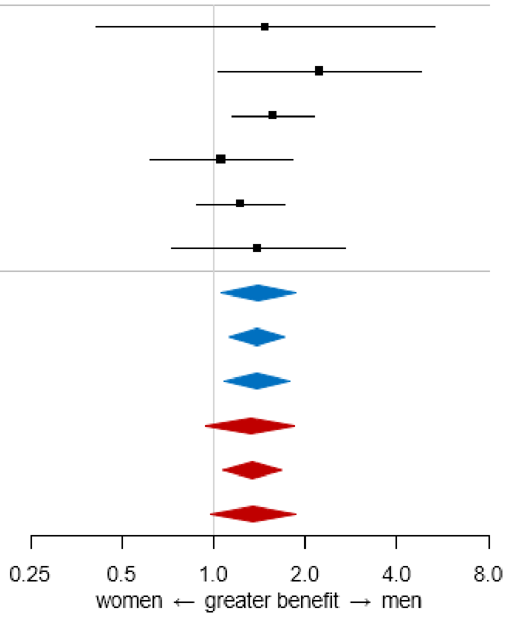

Supplementary Figure 5: Analysis of interaction between age and iv iron effects on the composite endpoint of total (first and recurrent) heart failure hospitalizations and cardiovascular mortality. Shown are the ratios of rate ratios (RRRs) comparing the treatment effects in each study's two subgroups (age <69.4 vs. ≥69.4 years) (p_B=0.235, I^2=29%).

study	N	RRR	95% CI
FAIR−HF	459	0.66	[0.10, 4.54]
CONFIRM−HF	301	0.61	[0.14, 2.55]
AFFIRM−AHF	1108	0.61	[0.38, 0.99]
IRONMAN	1137	0.79	[0.45, 1.40]
HEART−FID	3065	1.04	[0.78, 1.40]
FAIR−HF2	1105	0.89	[0.53, 1.49]
**overall (6 studies >200 pat., HN(0.5))**		**0.84**	**[0.59, 1.16]**
overall (6 studies, HN(0.1))		*0.86*	*[0.68, 1.08]*
overall (6 studies, KnHa)		*0.87*	*[0.67, 1.14]*
overall (4 studies >1000 pat., HN(0.5))		0.86	[0.58, 1.22]
overall (4 studies, HN(0.1))		*0.87*	*[0.69, 1.10]*
overall (4 studies, KnHa)		*0.87*	*[0.60, 1.26]*

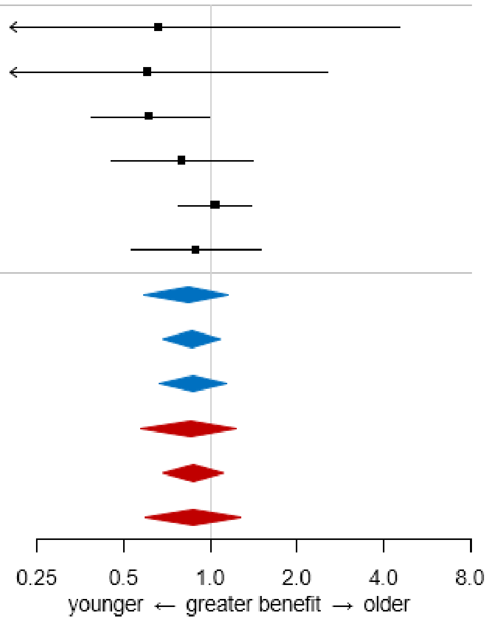

Supplementary Figure 6: Analysis of interaction between HF etiology and iv iron effects on the composite endpoint of total (first and recurrent) heart failure hospitalizations and cardiovascular mortality. Shown are the ratios of rate ratios (RRRs) comparing the treatment effects in each study's two subgroups (ischemic vs. non-ischemic, including unknown) (p_B=0.273, I^2=36%).

study	N	RRR	95% CI
FAIR−HF	459	2.07	[0.20, 21.10]
CONFIRM−HF	301	0.80	[0.16, 3.96]
AFFIRM−AHF	1108	0.55	[0.34, 0.87]
IRONMAN	1137	0.86	[0.55, 1.34]
HEART−FID	3065	0.84	[0.62, 1.13]
FAIR−HF2	1105	1.26	[0.72, 2.21]
**overall (6 studies >200 pat., HN(0.5))**		**0.84**	**[0.59, 1.22]**
overall (6 studies, HN(0.1))		*0.83*	*[0.66, 1.04]*
overall (6 studies, KnHa)		*0.83*	*[0.61, 1.12]*
overall (4 studies >1000 pat., HN(0.5))		0.82	[0.55, 1.25]
overall (4 studies, HN(0.1))		*0.82*	*[0.65, 1.04]*
overall (4 studies, KnHa)		*0.82*	*[0.50, 1.36]*

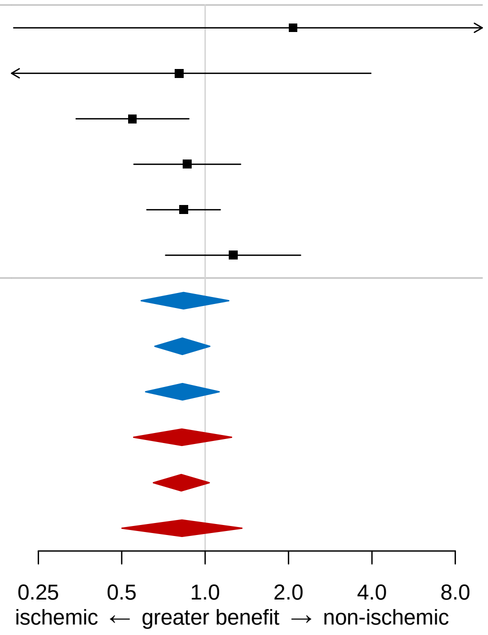

Supplementary Figure 7: Analysis of interaction between baseline transferrin saturation (TSAT) and iv iron effects on the composite endpoint of total (first and recurrent) heart failure hospitalizations and cardiovascular mortality. Shown are the ratios of rate ratios (RRRs) comparing the treatment effects in each study's two subgroups (TSAT <20% vs. ≥20%) (p_B=0.253, I^2=14%).

study	N	RRR	95% CI
FAIR−HF	459	0.26	[0.03, 2.18]
CONFIRM−HF	301	0.38	[0.06, 2.19]
AFFIRM−AHF	1108	0.89	[0.47, 1.68]
IRONMAN	1137	0.83	[0.49, 1.41]
HEART−FID	3065	0.89	[0.66, 1.20]
FAIR−HF2	1105	0.95	[0.51, 1.77]
**overall (6 studies >200 pat., HN(0.5))**		**0.85**	**[0.61, 1.16]**
overall (6 studies, HN(0.1))		*0.86*	*[0.67, 1.09]*
overall (6 studies, KnHa)		*0.86*	*[0.65, 1.15]*
overall (4 studies >1000 pat., HN(0.5))		0.89	[0.64, 1.23]
overall (4 studies, HN(0.1))		*0.89*	*[0.69, 1.13]*
overall (4 studies, KnHa)		*0.89*	*[0.62, 1.27]*

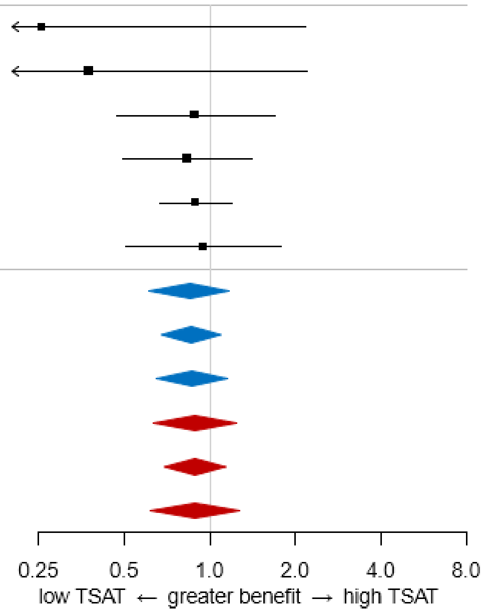

Supplementary Figure 8: Analysis of interaction between estimated glomerular filtration rate (eGFR) and iv iron effects on the composite endpoint of total (first and recurrent) heart failure hospitalizations and cardiovascular mortality. Shown are the ratios of rate ratios (RRRs) comparing the treatment effects in each study's two subgroups (eGFR ≤60 vs. >60 mL/min/1.73m²) (p_B=0.779, I²=19%).

study	N	RRR	95% CI
FAIR−HF	459	0.68	[0.07, 6.52]
CONFIRM−HF	301	0.95	[0.27, 3.28]
AFFIRM−AHF	1108	1.27	[0.73, 2.18]
IRONMAN	1137	0.79	[0.47, 1.33]
HEART−FID	3065	0.87	[0.64, 1.20]
FAIR−HF2	1105	1.14	[0.66, 1.95]
**overall (6 studies >200 pat., HN(0.5))**		**0.96**	**[0.70, 1.32]**
overall (6 studies, HN(0.1))		*0.96*	*[0.76, 1.21]*
overall (6 studies, KnHa)		*0.95*	*[0.72, 1.26]*
overall (4 studies >1000 pat., HN(0.5))		0.97	[0.68, 1.40]
overall (4 studies, HN(0.1))		*0.96*	*[0.75, 1.22]*
overall (4 studies, KnHa)		*0.95*	*[0.67, 1.37]*

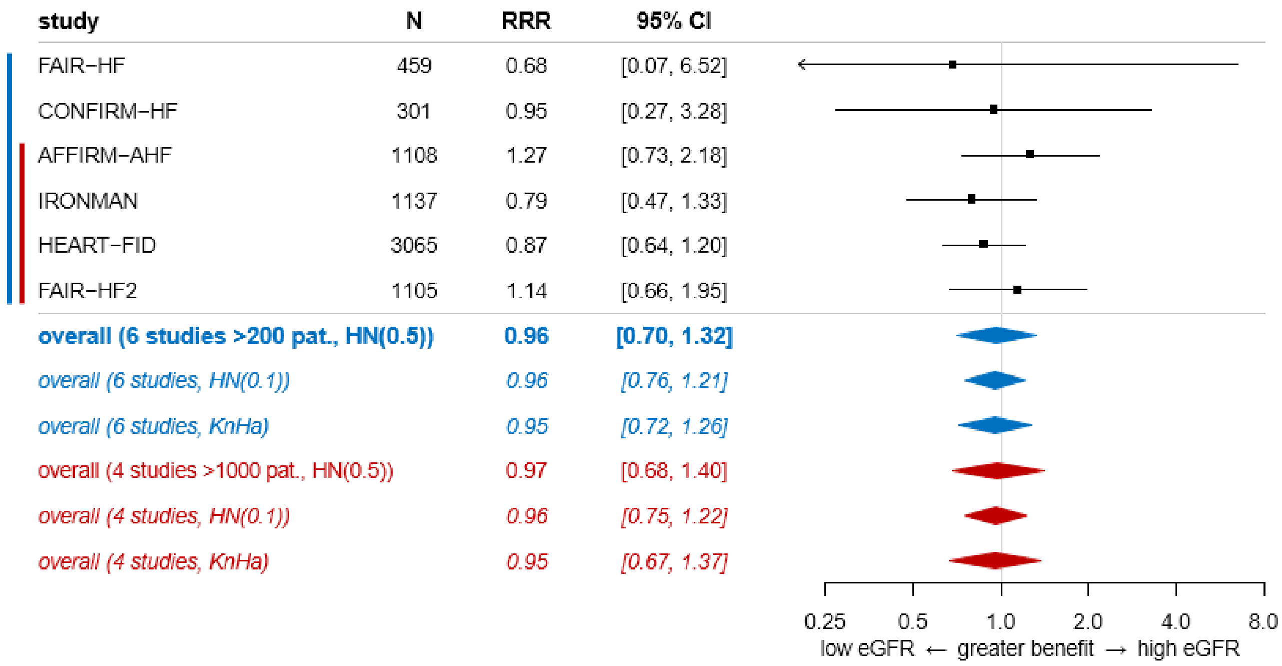

Supplementary Figure 9: Analysis of interaction between hemoglobin and iv iron effects on the composite endpoint of total (first and recurrent) heart failure hospitalizations and cardiovascular mortality. Shown are the ratios of rate ratios (RRRs) comparing the treatment effects in each study's two subgroups (hemoglobin <11.8 vs. ≥11.8 g/dL) (p_B=0.723, I^2=54%).

study	N	RRR	95% CI
FAIR−HF	459	0.63	[0.10, 4.08]
CONFIRM−HF	301	0.47	[0.13, 1.71]
AFFIRM−AHF	1108	1.26	[0.75, 2.12]
IRONMAN	1137	0.93	[0.60, 1.44]
HEART−FID	3065	0.69	[0.51, 0.93]
FAIR−HF2	1105	1.55	[0.93, 2.60]
**overall (6 studies >200 pat., HN(0.5))**		**0.94**	**[0.62, 1.43]**
overall (6 studies, HN(0.1))		*0.92*	*[0.73, 1.18]*
overall (6 studies, KnHa)		*0.95*	*[0.63, 1.43]*
overall (4 studies >1000 pat., HN(0.5))		1.00	[0.64, 1.66]
overall (4 studies, HN(0.1))		*0.95*	*[0.74, 1.23]*
overall (4 studies, KnHa)		*1.01*	*[0.57, 1.81]*

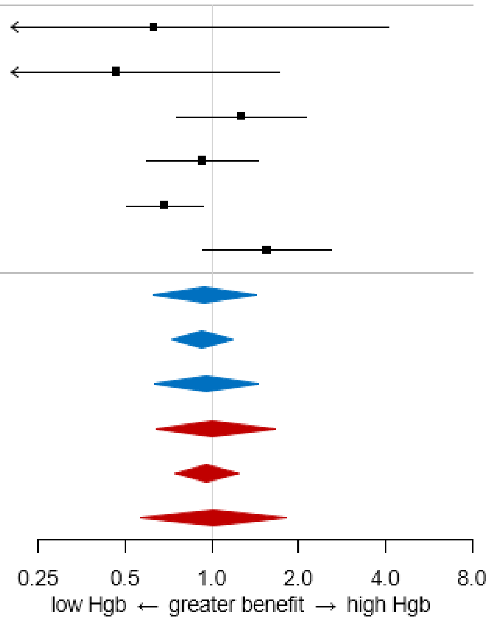

Supplementary Figure 10: Analysis of interaction between ferritin and iv iron effects on the composite endpoint of total (first and recurrent) heart failure hospitalizations and cardiovascular mortality. Shown are the ratios of rate ratios (RRRs) comparing the treatment effects in each study's two subgroups (ferritin <35 vs. ≥35 µg/l) (p_B=0.520, I²=57%).

study	N	RRR	95% CI
FAIR−HF	459	5.45	[0.45, 66.31]
CONFIRM−HF	301	2.51	[0.70, 8.94]
AFFIRM−AHF	1108	1.20	[0.65, 2.20]
IRONMAN	1137	0.97	[0.59, 1.59]
HEART−FID	3065	0.72	[0.53, 0.99]
FAIR−HF2	1105	1.75	[0.98, 3.10]
**overall (6 studies >200 pat., HN(0.5))**		**1.14**	**[0.74, 1.95]**
overall (6 studies, HN(0.1))		*1.02*	*[0.79, 1.35]*
overall (6 studies, KnHa)		*1.17*	*[0.69, 1.97]*
overall (4 studies >1000 pat., HN(0.5))		1.02	[0.65, 1.73]
overall (4 studies, HN(0.1))		*0.97*	*[0.74, 1.27]*
overall (4 studies, KnHa)		*1.04*	*[0.57, 1.90]*

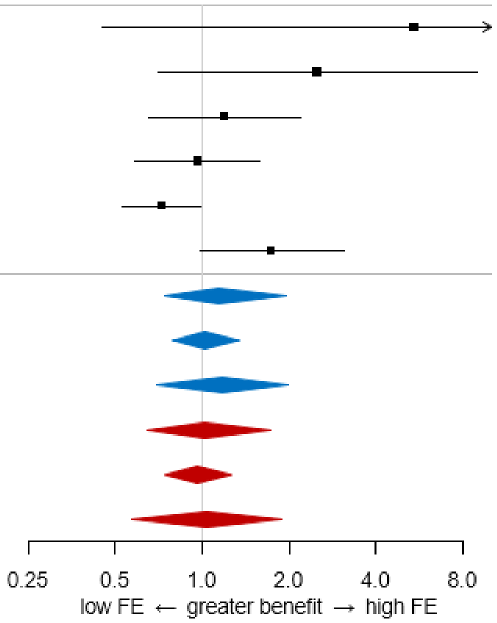

Supplementary Figure 11: Analysis of interaction between NYHA class and iv iron effects on the composite endpoint of total (first and recurrent) heart failure hospitalizations and cardiovascular mortality. Shown are the ratios of rate ratios (RRRs) comparing the treatment effects in each study's two subgroups (NYHA classes II vs. III-IV) (p_B=0.403, I^2=58%).

study	N	RRR	95% CI
CONFIRM−HF	301	1.13	[0.32, 3.96]
AFFIRM−AHF	1108	0.98	[0.62, 1.57]
IRONMAN	1137	0.81	[0.52, 1.26]
HEART−FID	3065	1.13	[0.84, 1.51]
FAIR−HF2	1105	0.50	[0.31, 0.81]
**overall (5 studies >200 pat., HN(0.5))**		**0.87**	**[0.57, 1.29]**
overall (5 studies, HN(0.1))		*0.89*	*[0.70, 1.12]*
overall (5 studies, KnHa)		*0.86*	*[0.57, 1.31]*
overall (4 studies >1000 pat., HN(0.5))		0.85	[0.53, 1.31]
overall (4 studies, HN(0.1))		*0.88*	*[0.69, 1.11]*
overall (4 studies, KnHa)		*0.84*	*[0.48, 1.47]*

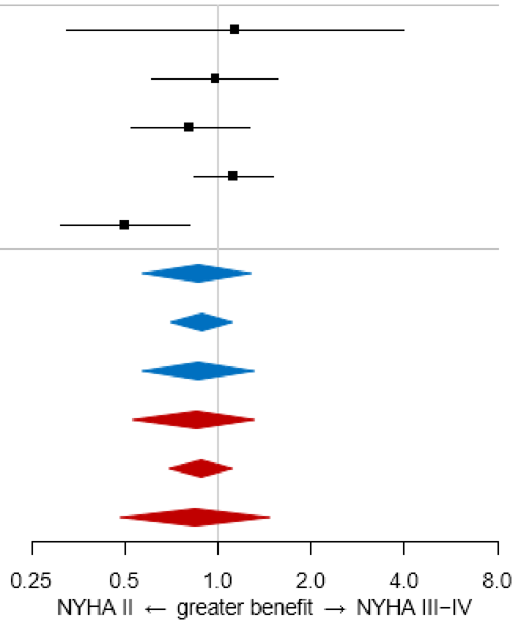

Supplementary Figure 12: The effect of intravenous iron on the composite endpoint of total (first and recurrent) heart failure hospitalizations and cardiovascular mortality with respect to men and women.

study	women (RR)	men (RR)
FAIR−HF	0.51 [0.13, 2.03]	0.43 [0.12, 1.56]
CONFIRM−HF	0.87 [0.31, 2.42]	0.39 [0.18, 0.84]
AFFIRM−AHF	1.00 [0.70, 1.42]	0.64 [0.47, 0.87]
IRONMAN	0.86 [0.53, 1.39]	0.81 [0.63, 1.04]
HEART−FID	1.07 [0.81, 1.42]	0.89 [0.75, 1.06]
FAIR−HF2	1.04 [0.58, 1.86]	0.79 [0.60, 1.05]
**overall**	**0.98 [0.75, 1.26]**	**0.76 [0.56, 0.95]**

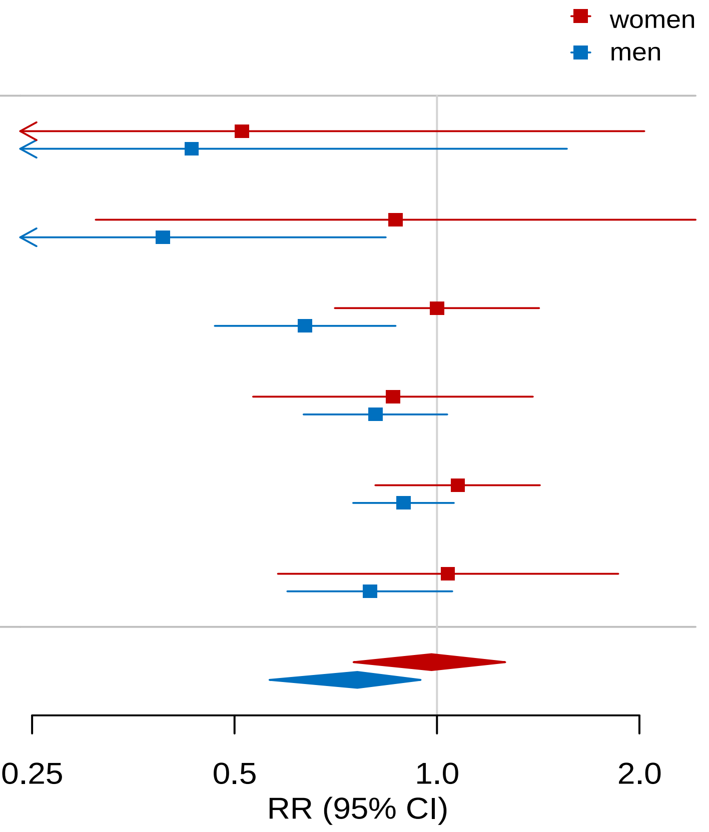

Heterogeneity (tau): 0.11 [0.00, 0.39]
Heterogeneity (tau): 0.15 [0.00, 0.46]

n / N: number of events / number of subjects in group

Supplementary Figure 13: Effect estimates based on sensitivity analyses using the primary endpoint of time to first event for cardiovascular mortality and HF hospitalization, with a half-normal prior of HN (0.5).

**study**	**HR**	**95% CI**
AFFIRM	0.80	[0.66, 0.97]
IRONMAN	0.84	[0.70, 1.01]
HEART−FID	0.93	[0.81, 1.06]
FAIR−HF2	0.77	[0.62, 0.97]
**overall**	**0.85**	**[0.70, 1.01]**

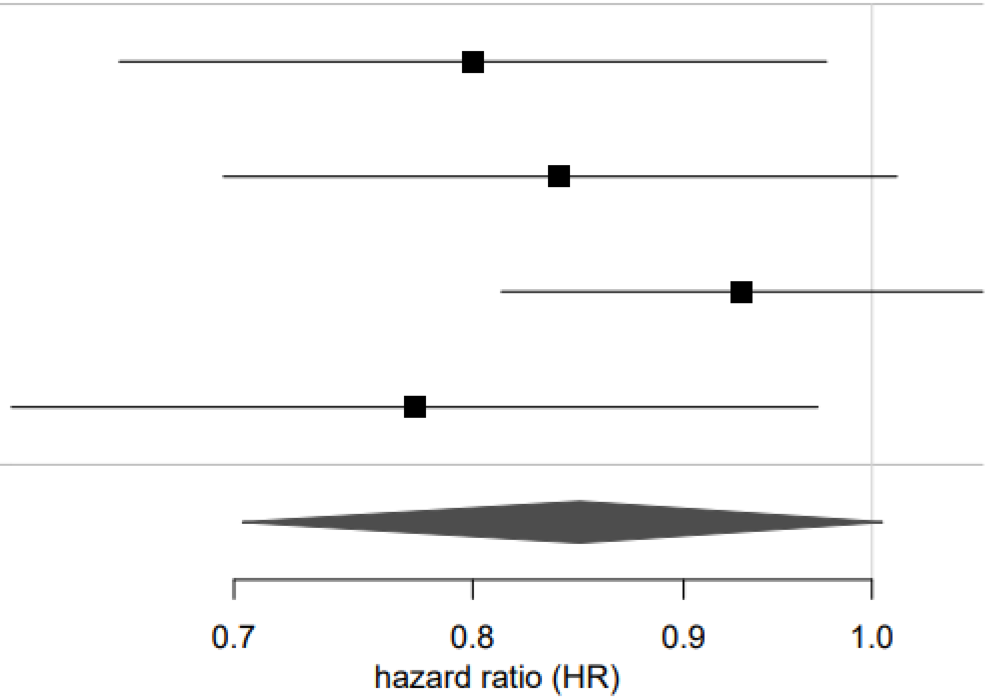

Heterogeneity (tau): 0.08 [0.00, 0.32]

Supplementary Figure 14: Effect estimates based on sensitivity analyses using the primary endpoint of time to first event for cardiovascular mortality and HF hospitalization, with a half-normal prior of HN (0.1).

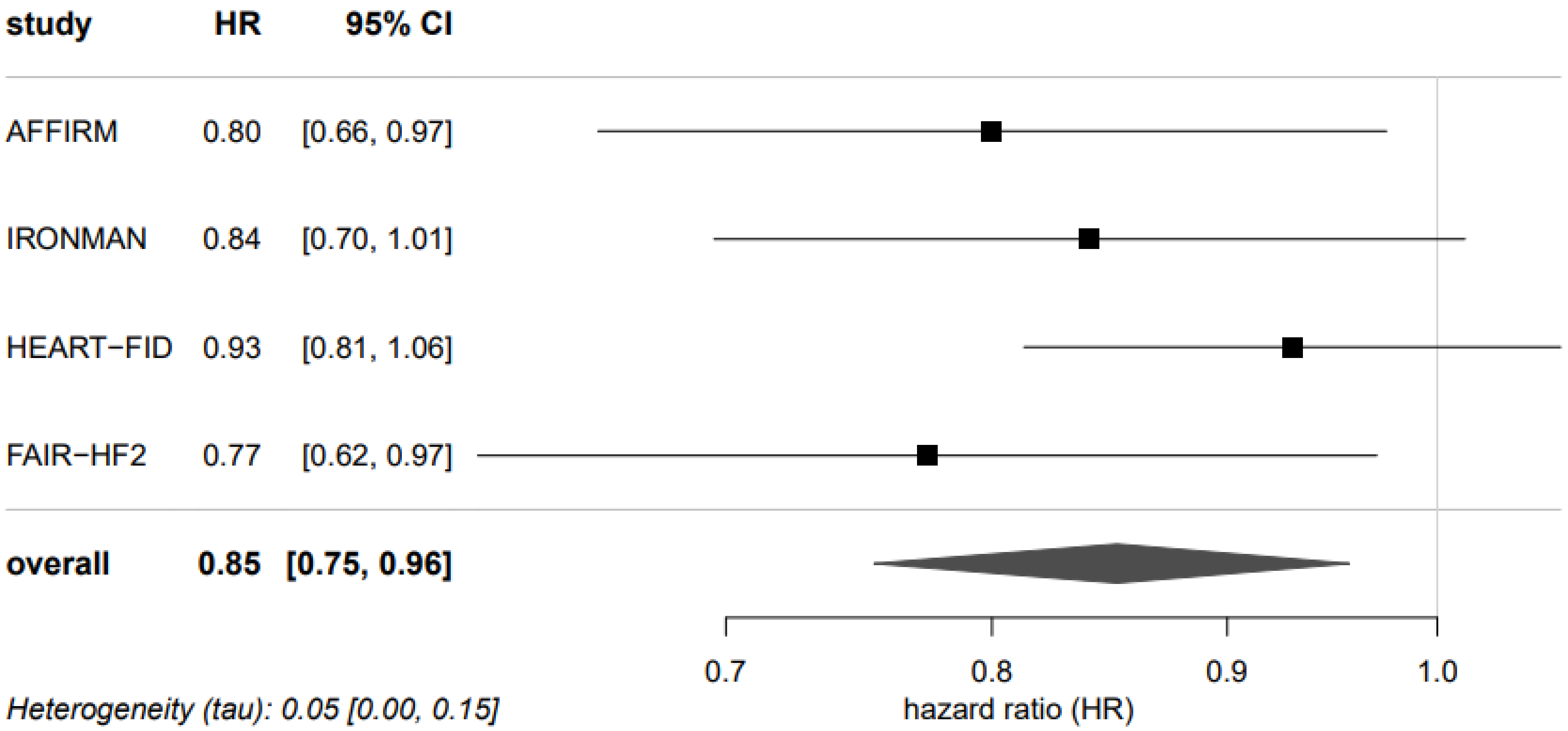

Supplement: Supplementary file 1 — Supplementary Tables 1 and 2 and Figs. 1–14. [file 41591_2025_3671_MOESM1_ESM.pdf]
