# Supplementary material for: Systematic review and meta-analysis of intravenous iron therapy for patients with heart failure and iron deficiency
Source: Nat Med. 2025 Mar 30;31(8):2640–6. doi: 10.1038/s41591-025-03671-1 (PMC12353798; doi:10.1038/s41591-025-03671-1)
Supplement: Supplementary file 2 — Reporting Summary [file 41591_2025_3671_MOESM2_ESM.pdf]

nature portfolio

Corresponding author(s): Stefan D. Anker MD, PhD

Last updated by author(s): Mar 16, 2025

Reporting Summary

Nature Portfolio wishes to improve the reproducibility of the work that we publish. This form provides structure for consistency and transparency in reporting. For further information on Nature Portfolio policies, see our Editorial Policies and the Editorial Policy Checklist.

Statistics

For all statistical analyses, confirm that the following items are present in the figure legend, table legend, main text, or Methods section.

n/a	Confirmed	
☐	☒	The exact sample size (*n*) for each experimental group/condition, given as a discrete number and unit of measurement
☒	☐	A statement on whether measurements were taken from distinct samples or whether the same sample was measured repeatedly
☐	☒	The statistical test(s) used AND whether they are one- or two-sided<br>*Only common tests should be described solely by name; describe more complex techniques in the Methods section.*
☐	☒	A description of all covariates tested
☐	☒	A description of any assumptions or corrections, such as tests of normality and adjustment for multiple comparisons
☐	☒	A full description of the statistical parameters including central tendency (e.g. means) or other basic estimates (e.g. regression coefficient) AND variation (e.g. standard deviation) or associated estimates of uncertainty (e.g. confidence intervals)
☐	☒	For null hypothesis testing, the test statistic (e.g. *F*, *t*, *r*) with confidence intervals, effect sizes, degrees of freedom and *P* value noted<br>*Give P values as exact values whenever suitable.*
☐	☒	For Bayesian analysis, information on the choice of priors and Markov chain Monte Carlo settings
☒	☐	For hierarchical and complex designs, identification of the appropriate level for tests and full reporting of outcomes
☒	☐	Estimates of effect sizes (e.g. Cohen's *d*, Pearson's *r*), indicating how they were calculated

Our web collection on statistics for biologists contains articles on many of the points above.

Software and code

Policy information about availability of computer code

Data collection	A comprehensive search of PubMed and Scopus was conducted, supplemented by a review of ClinicalTrials.gov, recent reviews, and meta-analyses. No software was used.
Data analysis	All analyses were performed using R (version 4.4 or higher) or SAS (version 9.4 or higher). Cochrane risk of bias tool was used to assess risk of biasness.

For manuscripts utilizing custom algorithms or software that are central to the research but not yet described in published literature, software must be made available to editors and reviewers. We strongly encourage code deposition in a community repository (e.g. GitHub). See the Nature Portfolio guidelines for submitting code & software for further information.

Data

Policy information about availability of data

All manuscripts must include a data availability statement. This statement should provide the following information, where applicable:

- Accession codes, unique identifiers, or web links for publicly available datasets
- A description of any restrictions on data availability
- For clinical datasets or third party data, please ensure that the statement adheres to our policy

The data will be made available to any researcher upon request by contacting the corresponding author within 2 to 4 weeks to ensure appropriate use and

interpretation.

Research involving human participants, their data, or biological material

Policy information about studies with human participants or human data. See also policy information about sex, gender (identity/presentation), and sexual orientation and race, ethnicity and racism.

Reporting on sex and gender	Subgroup analyses were conducted based on sex, as all included trials provided sex-specified data.
Reporting on race, ethnicity, or other socially relevant groupings	No anaylsis was conducted stratified by race or any other social groupings
Population characteristics	Adults with HF, iron deficiency , and an LVEF 50% or lower were included. Average age ranged from 67 to 74 years
Recruitment	Consenting patients meeting the inclusion criteria such as HF, iron deficiency, and LVEF<=50% were recruited. Only IRONMAN was open label trial.
Ethics oversight	The trials protocols were approved by an ethics committee. This meta-analysis is exempt from ethical approval

Note that full information on the approval of the study protocol must also be provided in the manuscript.

Field-specific reporting

Please select the one below that is the best fit for your research. If you are not sure, read the appropriate sections before making your selection.

☒ Life sciences ☐ Behavioural & social sciences ☐ Ecological, evolutionary & environmental sciences

For a reference copy of the document with all sections, see nature.com/documents/nr-reporting-summary-flat.pdf

Life sciences study design

All studies must disclose on these points even when the disclosure is negative.

Sample size	All six trials enrolled 7,175 patients including 3,672 randomized to IV iron and 3,503 to control groups
Data exclusions	Trials that did not include relevant populations or report outcomes of interest were excluded.
Replication	We obtained patient-level data from five trials and applied IRONMAN trial methods, available only at the trial level
Randomization	All six trials randomized patients to IV iron or placebo/usual care group.
Blinding	FIve trials were double-blind, while IRONMAN was open label; however, its primary endpoint was assessed through blinded outcome adjudication to minimize bias.

Reporting for specific materials, systems and methods

We require information from authors about some types of materials, experimental systems and methods used in many studies. Here, indicate whether each material, system or method listed is relevant to your study. If you are not sure if a list item applies to your research, read the appropriate section before selecting a response.

Materials & experimental systems

n/a	Involved in the study	
☒	☐	Antibodies
☒	☐	Eukaryotic cell lines
☒	☐	Palaeontology and archaeology
☒	☐	Animals and other organisms
☐	☒	Clinical data
☒	☐	Dual use research of concern
☒	☐	Plants

Methods

n/a	Involved in the study	
☒	☐	ChIP-seq
☒	☐	Flow cytometry
☒	☐	MRI-based neuroimaging

Clinical data

Policy information about clinical studies

All manuscripts should comply with the ICMJE guidelines for publication of clinical research and a completed CONSORT checklist must be included with all submissions.

Clinical trial registration	All trials are registered on clinicalTrials.gov. FAIR-HF2: NCT03036462; IRONMAN: NCT02642562; AFFIRM: NCT02937454; CONFIRM: NCT01453608; FAIR-HF: NCT00520780 and HEART-FID: NCT03037931.
Study protocol	The stduy protocol can be found on https://www.crd.york.ac.uk/prospero/ using PROSPERO Registration number: CRD42025635165.
Data collection	We obtained patient-level data for FAIR-HF, FAIR-HF2, CONFIRM-HF, AFFIRM-AHF, and HEART-FID trials and trial level data was obtained for IRONMAN trial.
Outcomes	The primary endpoint was the composite of recurrent HF hospitalizations or CV death. Secondary endpoints for this analysis included total HF hospitalizations, CV and all-cause mortality. Safety endpoints included serious adverse events or hospitalizations due to infections. Outcomes were assessed via clinical records, investigator reports, and independent adjudication using standardized definition

Plants

Seed stocks	*Report on the source of all seed stocks or other plant material used. If applicable, state the seed stock centre and catalogue number. If plant specimens were collected from the field, describe the collection location, date and sampling procedures.*
Novel plant genotypes	*Describe the methods by which all novel plant genotypes were produced. This includes those generated by transgenic approaches, gene editing, chemical/radiation-based mutagenesis and hybridization. For transgenic lines, describe the transformation method, the number of independent lines analyzed and the generation upon which experiments were performed. For gene-edited lines, describe the editor used, the endogenous sequence targeted for editing, the targeting guide RNA sequence (if applicable) and how the editor was applied.*
Authentication	*Describe any authentication procedures for each seed stock used or novel genotype generated. Describe any experiments used to assess the effect of a mutation and, where applicable, how potential secondary effects (e.g. second site T-DNA insertions, mosiacism, off-target gene editing) were examined.*
